# Exploring mobility in Bronze Age Greece: A Sr isotope perspective

**DOI:** 10.1371/journal.pone.0357576

**Published:** 2026-09-23

**Authors:** Anja B. Frank, Ioanna Moutafi, Serena Sabatini, Robert Frei, Sofia Voutsaki, Raphaël Orgeolet, Vassilis Lambrinoudakis, Lazaros Kolonas, Jörg Rambach, Michael Boyd, Evi Margaritis, Kristian Kristiansen, Karin M. Frei

**Affiliations:** 1 Department of Research, Collections and Conservation, Environmental Archaeology and Materials Science, National Museum of Denmark, Kongens Lyngby, Denmark; 2 Institute for Pre- and Protohistoric Archaeology and Archaeology of the Roman Provinces, Ludwig-Maximilians-Universität München, Munich, Germany; 3 The Malcolm H. Wiener Laboratory for Archaeological Science, American School of Classical Studies at Athens, Athens, Greece; 4 Department of Historical Studies, University of Gothenburg, Gothenburg, Sweden; 5 Department of Geosciences and Natural Resource Management, University of Copenhagen, Copenhagen, Denmark; 6 Groningen Institute of Archaeology, University of Groningen, Groningen, Netherlands; 7 Aix Marseille University, CNRS, CCJ, Aix-en-Provence, France; 8 Department of History and Archaeology, National and Kapodistrian University of Athens, Athens, Greece; 9 Ministry of Culture, Athens, Greece; 10 German Archaeological Institute, Athens, Greece; 11 British School at Athens, Athens, Greece; 12 Science and Technology in Archaeology and Culture Research Centre (STARC), The Cyprus Institute, Nicosia, Cyprus; 13 Globe Institute, Lundbeck Foundation GeoGenetics Centre, Copenhagen, Denmark; Austrian Academy of Sciences, AUSTRIA

## Abstract

The Mediterranean region saw significant socio-economic and cultural transformations during the Bronze Age, varying across regions and through time. Expanding exchange networks played an important role in these developments by connecting distant communities. To what extent these exchanges were accompanied by the migration and settlement of foreign individuals within local communities is still poorly understood. Here we present Sr isotope data from 199 skeletal samples belonging to individuals excavated at six archaeological sites from central and southern Greece to explore how mobility changed throughout the Bronze Age and whether it was driven by specific factors. In order to identify foreign individuals to the investigated sites we further established site specific Sr isotope baselines for their surrounding area based on published data as well as for the sites themselves based on the ⁸⁷Sr/⁸⁶Sr signatures of the buried individuals themselves. The comparison of the individuals’ ⁸⁷Sr/⁸⁶Sr signatures to respective baselines revealed 17 non-locals, all of which could have come from other parts of Greece. Migration or mobility towards communities in central and southern Greece likely appeared at a fairly consistent rate throughout the Bronze Age as no more than 5% − 10% non-locals could be identified throughout different stages of the Bronze Age. The non-locals included individuals of both genders, revealing no clear gender biases on mobility, while the approximate age of the identified non-locals indicates that individuals had most likely moved as young adults. It is therefore likely that mobility involved individuals rather than the resettlement of entire families. By linking individual Sr isotope data from multiple archaeological sites, this study reveals complex mobility patterns characterized by multidirectional movement driven by diverse cultural and economic factors.

## Introduction

During the Bronze Age, the region of modern-day Greece underwent substantial social, economic, and cultural transformations, differently expressed across regions and through time. In particular, the transition from the Middle to Late Bronze Age witnessed the formation of Mycenaean societies on the mainland, accompanied by increasing socio-political complexity, expanding exchange networks, and significant changes in settlement organisation and material culture. Following diverse local trajectories, these developments were crucial for the emergence of Mycenaean palatial societies and continued to shape communities during the palatial and post-palatial times of the Late Bronze Age. Archaeological evidence has revealed major changes to mortuary practices, domestic architecture and settlement organisation [1], while recent and ongoing genetic studies suggest an increasingly complex demographic history, including the arrival of populations carrying Steppe-related ancestry beginning around 2200 BC [2–5]. However, patterns of individual mobility within local communities remain poorly understood. Exchange of goods, ideas and cultural practices is well attested across the Mediterranean and the Aegean throughout the Bronze Age, although its intensity and character varied across regions and periods [6], but to what degree this was accompanied by migration of foreign individuals to local communities has not been thoroughly investigated yet. Indeed, archaeological evidence fails to determine whether goods and ideas were introduced by traders, which never entered the local community, or through individual migration and settlement to the local community. Although ancient DNA studies have recently revealed otherwise undetected evidence of past mobility by identifying Anatolian ancestry for prehistoric individuals from the Greek Island Euboea [5], bioarchaeological research on the question of human mobility during this period is still fairly limited [7].

In the last decades, Sr isotope investigations of archaeological remains have opened a window to determine past mobility on an individual level enabling the identification of non-local individuals within an archaeological community [8,9]. As ⁸⁷Sr/⁸⁶Sr does not fractionate significantly from soil to crop, to the incorporation within the skeleton of an individual [10], it is possible to determine whether an individual migrated to the site they were buried at by comparing their skeletal Sr isotope signatures to the bioavailable ⁸⁷Sr/⁸⁶Sr signature of the excavation site and surrounding area. In recent years, multiple studies have been published on the bioavailable Sr isotope baseline of Greece or parts thereof [11–13], providing a solid base for investigating past individual mobility. However, despite Greece’s rich Bronze Age history, only a few Sr-based Bronze Age mobility studies exist to date [14–17], most of which focus on a single context or only a handful of individuals. Hence, individual mobility within the Bronze Age population of mainland modern-day Greece is still largely unexplored, and, furthermore, our current understanding is heavily biased to specific sites, emphasising the need for more large-scale Sr isotope studies from different archaeological sites in Greece.

**Fig 1:**
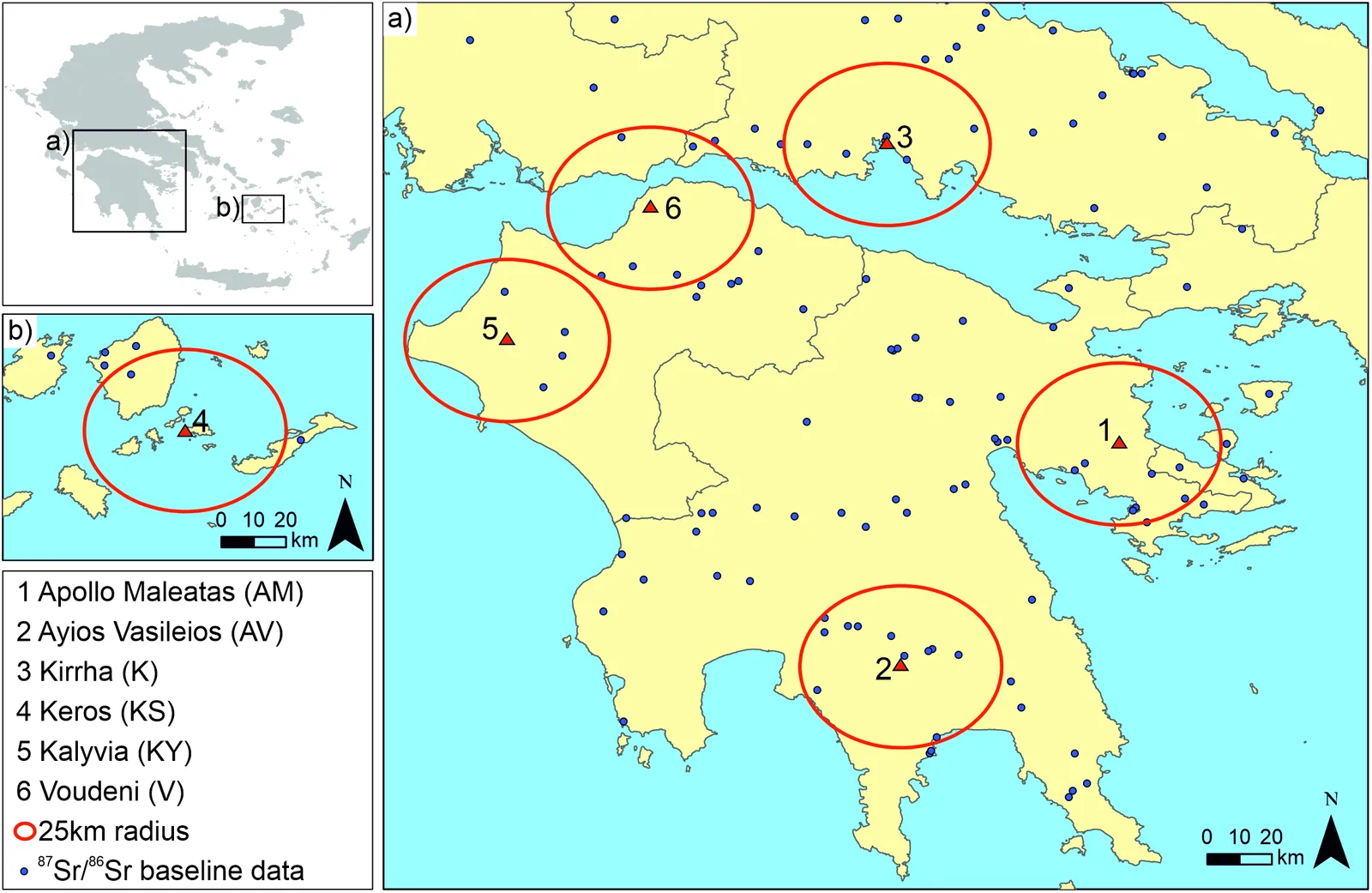
Locations of the investigated archaeological sites in modern-day Greece. The red circles indicate the 25 km radius around the sites used to calculate their baselines, while the blue dots are locations for which bioavailable ⁸⁷Sr/⁸⁶Sr baseline data has been reported before [11–13,15,18,19]. The country outline for modern-day Greece was sourced from GADM [20].

This study presents human ⁸⁷Sr/⁸⁶Sr isotope data from six archaeological sites from central and southern Greece to determine the presence of non-locals as well as potential migratory patterns and pathways during the Bronze Age (Fig 1). Most of the investigated individuals were excavated from three main archaeological sites, Kirrha, Ayios Vasileios, and Voudeni. The first two sites date from the Middle Bronze Age (MBA) to Late Bronze Age (LBA) transition, while the latter dates to the LBA, placing the main focus of this study on individual mobility within these periods. However, several individuals from two Early Bronze Age (EBA) sites (Apollo Maleatas and Keros) and one Neolithic cemetery (Kalyvia) were also included and give an idea of how mobility may have evolved throughout the Bronze Age.

## Archaeological sites

### Apollo maleatas

The site of Apollo Maleatas at Mount Kynortion, Epidavros, Argolid, is primarily recognized for the Archaic and Classical sanctuary of Apollo Maleatas situated on the northern slope of the hill. At the summit of Mount Kynortion, a small settlement dating to the Early Bronze Age was identified [21]. Following the abandonment of this settlement around the end of the Early Helladic (EH) period, certain activities—possibly ritual in nature—are attested in the area and dated to the Middle Helladic (MH) period [22]. During the Mycenaean and Geometric times, ritual practices continued (located gradually further down the hillside) and became more intensive in historical times with the establishment of Apollo Maleatas Sanctuary.

Three burials (Graves 1–3) were discovered on the northeastern edge of the hilltop, next to the EH buildings. They consisted of individual interments placed in pits and covered with coarse limestone slabs. The deceased were laid on their sides in contracted positions. Grave 1 contained no burial offerings, whereas the other two included grave goods (mainly tools and personal ornaments). Additionally, fragments of pottery and tools were deposited outside Grave 2. Initially, the burials were dated to the EH I period due to their association with the earliest phase of occupation on the hill. However, radiocarbon analysis has dated all three burials to the 19th century BCE, placing them within the MH period.

### Ayios Vasileios

Ayios Vasileios, the site of the recently identified Mycenaean palace dating to the Late Bronze Age (c. 1700–1100 BCE), is situated in Laconia about 12 km south of the modern city of Sparta. Excavations have been carried out there since 2010 by the Archaeological Society at Athens under the direction of the former Ephor of Antiquities, A. Vasilogamvrou. The site had already been inhabited during the Early Bronze Age (2nd millennium BCE), when a small village occupied the hilltop. This settlement appears to have been abandoned for much of the Middle Bronze Age before being reoccupied toward the end of that period, around 1700 BCE. A Late Helladic (LH) cemetery composed of stone-built graves (cists) and pits was found near the settlement, which is known as the North Cemetery. Its excavation took place between 2010 and 2016 under the supervision of S. Voutsaki from the University of Groningen. In total, 22 graves were uncovered (12 cists, 1 built tomb, and 9 pits), along with two “burials” consisting of small bone assemblages deposited above another grave [23].

The cemetery was used from the MH III to the LH II periods. Radiocarbon dating of most of the skeletons—carried out by the Centre for Isotope Research at the University of Groningen, the 14C Chrono Laboratory at Queen’s University Belfast, and the Keck Carbon Cycle AMS Laboratory at UCI—confirmed that the cemetery dates between 1700 and 1450 BCE [24]. The graves at Ayios Vasileios largely continue earlier MH traditions, while also introducing new elements associated with the Mycenaean period, such as more elaborate cist constructions and a built tomb intended for multiple burials. Burial practices within the cemetery combine traditional single primary interments with newer practices, including collective depositions of successive burials and the presence of both primary and secondary interments within the same grave. The total MNI (=minimum number of individuals) for the cemetery is approximately 80. Most graves contain between one and four burials, some slightly more, and one notable example (Built tomb 21) has an MNI exceeding 25. Individuals of both sexes and all age groups are represented, although infants are clearly underrepresented and appear to have received somewhat different funerary treatment [25].

### Kalyvia

The cemetery at Kalyvia is located in the region of Elis in Western Greece and was discovered in 2004 during a rescue excavation led by Jörg Rambach [26]. The site dates to the period between the Final Neolithic and the Early Bronze Age (c. 3400–2800 BCE) and includes 24 graves. Most of these are rock-cut chamber tombs, although a small number of associated pit graves were also identified. The graves contained relatively modest grave goods, primarily ceramic vessels. This cemetery together with Kalamaki in Western Achaea represent the only cemeteries from this period so far discovered in the Peloponnese.

The chamber-tomb graves comprise a dromos (entrance passage), a stomion (entrance), and a burial chamber. Such architectural features represent an innovation for this period and were evidently designed to accommodate multiple interments. The chamber tombs—unlike the pit graves—were used repeatedly for successive burials and contained both primary and secondary skeletal deposits. Each tomb held between two and sixteen individuals, including adults and sub-adults of both sexes. The total MNI is preliminarily estimated around 150, but the osteological remains have not yet been analyzed.

### Keros

Keros, the focus of long-term excavations by the British School at Athens and the Hellenic Ministry of Culture, is a prehistoric site (ca. 2750−2250BCE) of utmost importance for the Early Bronze Age Aegean [27]. Dhaskalio Kavos along with the islet of Dhaskalio, on the western edge of Keros, is the island’s primary archaeological site, comprising: a) two “special deposits”, north and south, containing great concentrations of broken figurines, marble vessels, and ceramics, b) Kavos Promontory, bearing evidence of primary metallurgical activities (copper smelting) and obsidian production, c) a few burials of human remains in rock shelters to the south of the Special Deposit South, and d) the dense complex of buildings covering the (now) islet of Dhaskalio, which at that time was joined to Kavos by a narrow strip of land. Dhaskalio’s impressive architectural complex shows extensive evidence of large-scale metallurgical activities, although its exact character remains unclear. On Kavos, in contrast, the unique assemblages of fragmented marble figurines and other objects found in the “special deposits” have been interpreted as remains of ritual actions in an early pan-Cycladic maritime sanctuary, where people from dispersed communities gathered together to stress a common identity [27–29].

The human remains included in this study derive from two rock-shelters at Kavos (Area A) and date to the later phases of the Early Cycladic II period (ca. 2500–2200 BCE). The assemblage does not represent conventional burials: it consists of highly fragmented, disarticulated, and commingled bones, with no complete skeletons preserved. A minimum number of 13 individuals (MNI) is documented, including both sexes and all age categories. Bioarchaeological analysis revealed a highly unusual Cycladic funerary practice. Bodies were initially placed as primary burials in the rock-shelters. After decomposition, the skeletons underwent secondary rites: most bones were systematically removed elsewhere, leaving only small elements and teeth behind. This treatment was applied to all individuals except infants [30,31].

### Kirrha

Kirrha (Phokis, Central Greece), occupies a low mound today located roughly 230 m north of the Corinthian Gulf shoreline, near the modern town of Itea. The site was first explored by the French School at Athens in the late 1930s. These early investigations exposed large parts of a settlement sequence extending from the Middle Helladic period, ca. 2100–1700 BCE, into the Late Helladic period, ca. 1700–1075 BCE, and also brought to light numerous graves belonging to the same general span. Once these excavations had been published [32], Kirrha quickly became a major point of reference for Aegean prehistory and, more specifically, an essential site for the study of the Middle Helladic period in Central Greece.

At least 40 funerary contexts have been recorded at Kirrha, encompassing a range of burial forms such as stone-built cists, pits, a small number of shaft graves, and a few jar burials. In the Middle Helladic phases, burial is intramural and most often involves single individuals. A marked change is visible, however, in the transition from MH III to LH I/II, when funerary use extended over abandoned Middle Helladic houses and the mortuary record became more varied. This later horizon includes both single and multiple burials, involving men and women as well as individuals from all age groups, deposited in different grave constructions and according to different funerary treatments [33]. Large collective shaft graves of the later Mycenaean period are also known, although their precise relationship to the settlement remains to be established. The MNI is certainly above 80, but the final figure cannot yet be established since the osteological assemblage is still under study.

### Voudeni

The Mycenaean site of Voudeni is situated in western Achaea, about 7 km northeast of the modern city of Patras. The site includes a cemetery along with its associated settlement, although the settlement itself has only been minimally excavated. The cemetery was first discovered in 1987 and has since been systematically investigated by the local Ephorate of Antiquities under the supervision of Honorary General Director of Antiquities Lazaros Kolonas. Voudeni represents one of the largest Mycenaean cemeteries in the region of Achaea and remained in use for more than three centuries, spanning the entire period from the end of LH IIB to the LH IIIC/Sub-Mycenaean phase, approximately 1450–1050 BCE. The cemetery contains more than 80 chamber tombs, about 75% of which have been completely excavated. Thirty-five chamber tombs (Tombs 1–44) have been fully published by the excavator [34], and the bioarchaeological analysis from 20 of them has been conducted by Ioanna Moutafi [35]. Research on the remaining tombs is still ongoing.

The chamber tombs were constructed to accommodate multiple successive burials, and many remained in use throughout the full duration of the cemetery’s activity. The MNI per tomb ranges from 2 to 27, with an average of 10.3. Individuals of both sexes and all age groups are represented in the tombs, although adult burials predominate. In line with typical Mycenaean funerary customs, the tombs contain exclusively inhumations arranged as both primary and secondary burial deposits, usually accompanied by offerings (mostly pottery, but also jewelry, other personal ornaments, weapons, etc). Mortuary practices vary considerably, with emphasis shifting between individual and collective deposits, depending both on specific contexts and period of use.

## Materials and methods

### Material

A total of 199 inhumated Bronze Age individuals from six archaeological sites in central and southern Greece (Fig 1) were investigated for their childhood origin (The archive IDs for each sample are listed in S1 Table). Of these, 55 had been previously analysed for Sr isotopes [2]. The use of this published data is indicated in the sample overview (S1 Table). From each individual either a petrous bone (n = 92) or tooth enamel sample (n = 107) was analysed for Sr isotopes, enabling the determination of a potential foreign childhood origin, but not a mobility timeline for the individuals. The human teeth form in a well-established sequence, and their enamel portion does not remodel during an individual’s life or post mortem [8]. Thus, tooth enamel is a reliable archive of an individual’s diet and geographic origin during the crown’s formation making teeth the preferred sample type for this study. Wherever possible, sampling of the first molar was done (n = 52), as its formation commences around an individual’s birth and completes around three years of age, providing a clearly constrained ^87^Sr/^86^Sr signature of an individual’s early childhood. While bone samples are generally considered less suitable to determine childhood origin, as they are susceptible to alteration, the petrous bone has been shown to be resistant to remodelling and diagenetic alteration after the age of two [36,37]. Hence, they were considered a suitable Sr isotope archive of an individual’s early childhood, when no suitable teeth were available. Which specific tooth was chosen, or whether a petrous bone was sampled depended ultimately on the preservation of the remains, with altered looking or fragmented samples excluded from this study.

The archaeological material included in this study was provided by the excavation directors of each site. The archive IDs for all samples are listed in S1 Table. The permits required for this study were granted by the Greek Ministry of Culture and the study complied with all relevant regulations.

### Sex and age determination

The biological sex and approximate age-at-death of the investigated individuals given in S1 Table were determined through osteological analysis. Adult sex was estimated as male (including probable male), indeterminate, and female (including probable female) on the basis of sexually dimorphic morphological characteristics of the skull and the pelvis, following well-established protocols [38]. Juvenile remains were excluded from sex estimation due to the current lack of reliable techniques. Adult age-at-death was estimated from morphological alterations on the pelvis, degree of cranial suture closure, and dental wear [38]. Non-adult age-at-death was estimated based on level of dental development [39,40], long bone diaphyseal length, and degree of epiphyseal union [41]. Age categories as listed in S1 Table were sub-divided in simplified categories (sub-adult: birth to 18 years, young adult: 18–30 years, adult: 30 years and older). Additionally, the genetic sex could be verified for selected individuals by aDNA analysis [2].

### Sr isotope analysis

The skeletal samples were taken using a diamond-tipped drill. The outer layer of the samples was removed to avoid any contamination and 1–2 mg of clean enamel or dense petrous bone were cut from the tooth and bone samples, respectively. Using a 1:1 solution of 0.5 ml 6M HCl and 0.5 ml 30% H_2_O_2_ the samples were dissolved. The bone samples were spiked with a ^84^Sr enriched tracer to determine Sr concentration via isotope dilution (ID), before all samples were dried down on a hot plate at 100°C.

The Sr column separation followed largely the methods used by [42]. Pre-cleaned, press-in filters were fitted into 1 ml pipette tips to serve as disposable extraction columns, which were charged using 200 µl pre-cleaned SrSpec™ resin (50–100 mesh; Eichrome Inc./Tristchem). The columns were conditioned with 3M HNO_3_. The prepared samples were re-dissolved in a few drops of 3M HNO_3_ and loaded onto the columns. After washing the samples with ~10 ml of 3M HNO_3_, Sr was collected using 2 ml of MQ and dried down on a hot plate at 100°.

The Sr concentrations and isotope compositions were determined at the University of Copenhagen using a VG Sector 54 IT mass spectrometer equipped with eight Faraday detectors. The standard reference material SRM 987 was run repeatedly between samples sets resulting in an average ^87^Sr/^86^Sr ratio of 0.710239 ± 0.00002 (n = 8, 2σ). The measured ^87^Sr/^86^Sr values of the samples were corrected for the slight offset to the published mean SRM 987 value (0.710245) [43].

### Sr isotope baseline calculation

In order to calculate site-specific bioavailable Sr isotope baselines, we utilized available ^87^Sr/^86^Sr data from central and southern Greece. A 25 km radius was drawn around the investigated archaeological sites, as this was considered the most likely range local food was sourced from (Fig 1), and the published bioavailable Sr isotope data within these areas was compiled for each site. The data included various proxies for bioavailable Sr, such as biomineral data from human or faunal remains [13,15,18] or environmental samples [11,12,19]. Some studies reported multiple ^87^Sr/^86^Sr values at the same location [2,6]. As a result, these were averaged to avoid an overrepresentation of this location in the dataset. Based on this, between 1 and 11 baseline data points were compiled for the investigated archaeological sites (Table 1). Using this data, baselines were defined as the average bioavailable ⁸⁷Sr/⁸⁶Sr signature of the area ± its double standard deviation (x̅ ± 2σ) for all sites, but Keros. The latter was represented by only 1 published bioavailable ^87^Sr/^86^Sr value within the 25 km radius, making it clear that a secondary approach was needed for baseline definition.

**Table 1:** Calculated bioavailable Sr isotope baselines for the investigated archaeological sites.

Abbreviation	Archaeological site	n baseline points	^87^Sr/^86^Sr avg	2SD	Lower limit	Upper limit	Data references
^87^Sr/^86^Sr baseline of the surrounding area within a radius of 25 km							
AM	Apollo Maleatas	7	0.70848	0.00055	0.70792	0.70903	[12,13,15]
AV	Ayios Vasileios	11	0.71119	0.00928	0.70190	0.72047	[12,15,18]
K	Kirrha	6	0.70835	0.00047	0.70788	0.70883	[11,19]
KS	Keros	1	0.70934				[13]
KY	Kalyvia	4	0.70853	0.00048	0.70806	0.70901	[12,19]
V	Voudeni	5	0.70829	0.00043	0.70786	0.70872	[11,12,19]
^87^Sr/^86^Sr baseline based on the potentially local excavated individuals							
AM	Apollo Maleatas	1	0.70872				this study
AV	Ayios Vasileios	51	0.70914	0.00079	0.70835	0.70993	this study
K	Kirrha	29	0.70862	0.00025	0.70837	0.70886	this study
KS	Keros	8	0.70936	0.00100	0.70837	0.71036	this study
KY	Kalyvia	3	0.70842	0.00044	0.70799	0.70886	this study
V	Voudeni	99	0.70838	0.00027	0.70811	0.70865	this study

To define secondary bioavailable Sr isotope baselines for the investigated archaeological sites, we utilized the human Sr isotope data presented in this study itself. Considering the direct link between the bioavailable Sr isotope composition of the human skeleton to its surroundings, this is a feasible approach, as long as the investigated sample set is large enough to represent the local population dynamic of the site. For example, an approach using all the human remains published to date within present-day Denmark, amounting to 628 human enamel and petrous bone Sr isotope signatures of individuals dating from the Neolithic to the Medieval, were used to statistically define a human-based Sr isotope range, which reliably characterizes the bioavailable Sr within Denmark through time [44]. This latter approach, in particular, was facilitated by the relatively homogeneous, glacial and country-wide surface geological circumstances met within present-day Denmark (island of Bornholm excluded). However, for sites represented by only a few individuals, such as Apollo Maleatas and Kalyvia, the presence of anomalous (non-local) individuals can result in a severely biased baseline. Hence, the human ⁸⁷Sr/⁸⁶Sr signature at each site were first screened for potential non-locals using the previously calculated baselines based on published data. Any individual falling out of these baselines were removed. Based on this, between 1 and 99 baseline datapoints were compiled for the investigated archaeological sites (Table 1). These were used to calculate site-specific baselines as the average bioavailable ⁸⁷Sr/⁸⁶Sr signature of the potential locals ± their double standard deviation (x̅ ± 2σ) for all sites, but Apollo Maleatas, as it has only one potentially local individual.

## Results

### Skeletal Sr data

The Sr isotope composition of all the skeletal remains reveal a comparatively narrow range from 0.7080 to 0.7105 (S1 Table). However, slight differences in the average and range of ⁸⁷Sr/⁸⁶Sr values could be observed between the investigated archaeological sites.

The lowest average ⁸⁷Sr/⁸⁶Sr signatures were recorded for Voudeni (n = 102) and Kalyvia (n = 3) with 0.7084 ± 0.0002 (x̄ ± 1σ) with the individuals from Kalyvia ranging from 0.7082 to 0.7087 and the individuals from Voudeni from 0.7080 to 0.7100. The individuals from Kirrha (n = 32) fall between 0.7084 and 0.7091, revealing the lowest standard deviation in ⁸⁷Sr/⁸⁶Sr values with a slightly higher average of 0.7086 ± 0.0001 (x̄ ± 1σ). The site of Apollo Maleatas (n = 3) returned individual ⁸⁷Sr/⁸⁶Sr signatures between 0.7087 and 0.7094 and an average of 0.7091 ± 0.0003 (x̄ ± 1σ). The individuals from Ayios Vasileios (n = 51) are characterised by a similar average ⁸⁷Sr/⁸⁶Sr value of 0.7091 ± 0.0004 (x̄ ± 1σ), but record a wider range in skeletal ⁸⁷Sr/⁸⁶Sr signatures from 0.7082 to 0.7101. Finally, the highest average ⁸⁷Sr/⁸⁶Sr value of 0.7094 ± 0.0005 (x̄ ± 1σ) was returned by the individuals excavated at Keros (n = 8), whose skeletal ⁸⁷Sr/⁸⁶Sr signatures ranged from 0.7089 to 0.7105.

The Sr concentration of the investigated bone samples showed a wide range from 62 mg/kg to 900 mg/kg (S1 Table). For the individuals from Voudeni and Kirrha, wide ranges in bone Sr concentration were measured between 64 mg/kg and 835 mg/kg (n = 60) and 147 mg/kg and 900 mg/kg (n = 16), respectively. The measured bone samples from Ayios Vasileios (n = 16) recorded Sr concentrations at the lower end of the spectrum with values between 62 mg/kg and 158 mg/kg. From Kalyvia only one bone sample was investigated with a Sr concentration of 116 mg/kg, while only teeth were analysed from Keros and Apollo Maleatas.

### Bioavailable Sr isotope data and baselines

**Fig 2:**
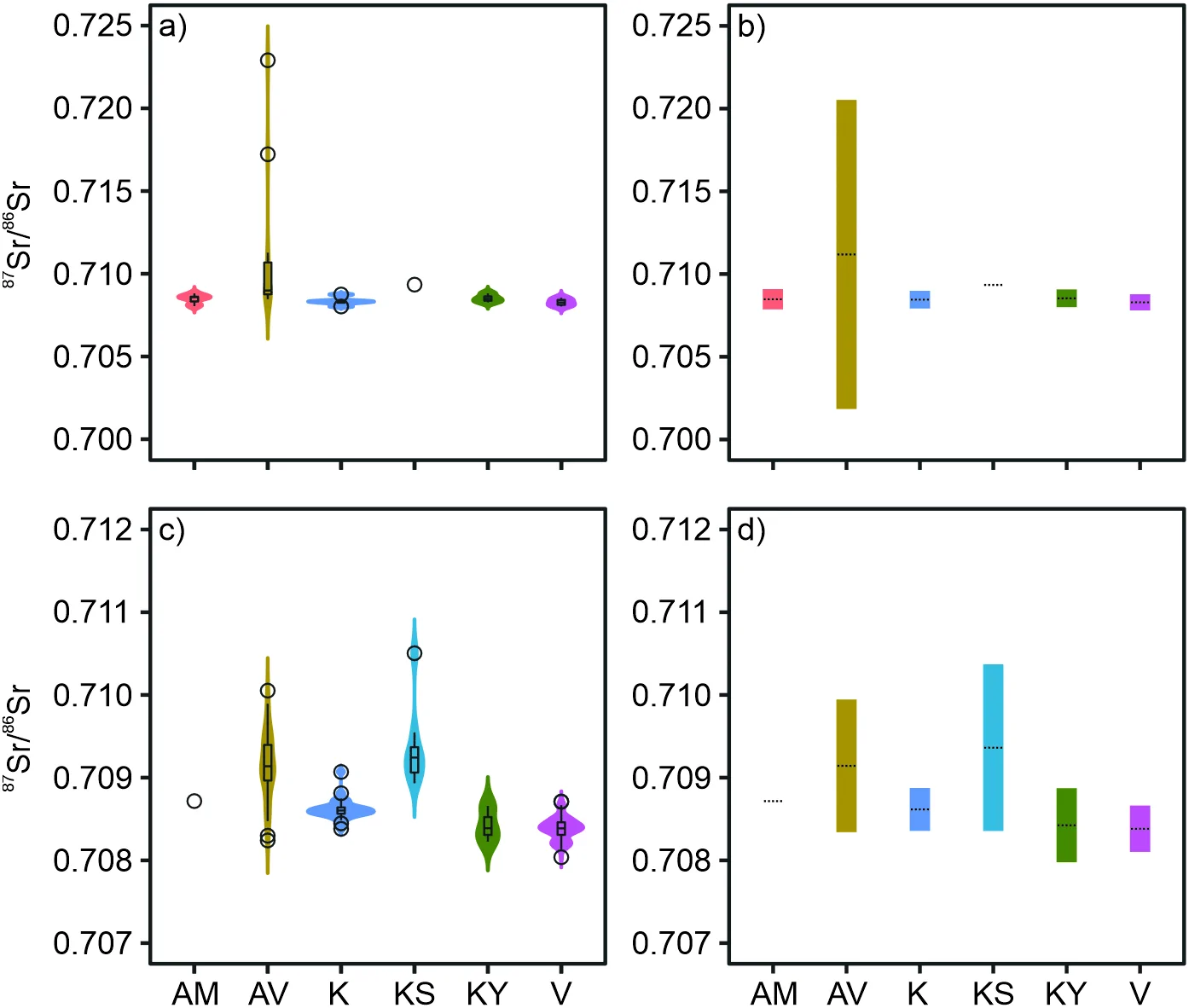
Bioavailable Sr data and calculated baselines for the investigated archaeological sites. a) Violin plot displaying the spread in proxy ^87^Sr/^86^Sr signatures published within a 25 km radius around the sites. b) Bioavailable Sr isotope baseline for the surrounding area of each site calculated as the average ^87^Sr/^86^Sr composition (dotted line) of the published data ± its double standard deviation (box). c) Violin plot displaying the spread in ^87^Sr/^86^Sr signatures of the potentially local individuals from each site. d) Bioavailable Sr isotope baseline for each site calculated as the average ^87^Sr/^86^Sr composition of the potential locals (dotted line) ± its double standard deviation (box). The abbreviations are as follows: AM – Apollo Maleatas, AV – Ayios Vasileios, K – Kirrha, KS – Keros, KY – Kalyvia, V – Voudeni.

The range of the compiled published bioavailable Sr isotope data within 25 km around the investigated sites showed a wide variability ranging from 0.7080 to 0.7229 (Fig 2a). The range in ⁸⁷Sr/⁸⁶Sr isotope signature reported for the surrounding area of Ayios Vasileios (n = 11) stands out, as it records a significantly wider range, in particular towards more radiogenic values, including the maximum ⁸⁷Sr/⁸⁶Sr signature. On the other hand, the surrounding areas of Apollo Maleatas (n = 7), Kirrha (n = 6), Kalyvia (n = 4) and Voudeni (n = 5) all record comparatively narrow ⁸⁷Sr/⁸⁶Sr ranges generally between 0.7080 to 0.7088. Only one baseline data point with a ⁸⁷Sr/⁸⁶Sr signature of 0.7093 is available within the vicinity of Keros, preventing the calculation of a meaningful Sr isotope baseline. The difference in reported bioavailable Sr isotope values around the investigated archaeological sites is reflected in their calculated baselines (Fig 2b, Table 1). The local ⁸⁷Sr/⁸⁶Sr baseline for Ayios Vasileios is defined as values between 0.7019 to 0.7205, while those of Apollo Maleatas, Kirrha, Kalyvia and Voudeni generally range from 0.7079 to 0.7090 with only slight variations.

After removing the skeletal ⁸⁷Sr/⁸⁶Sr signature of individuals that fall outside the previously defined baselines of the surrounding area, the remaining potentially local individual ⁸⁷Sr/⁸⁶Sr values were used to define a secondary set of site-specific baselines. The remaining skeletal ⁸⁷Sr/⁸⁶Sr values reveal different ranges in Sr isotope composition between the investigated archaeological sites (Fig 2c). From Apollo Maleatas, only one potentially local skeletal ⁸⁷Sr/⁸⁶Sr signature remains, not providing sufficient data for a baseline calculation. The range in expected ⁸⁷Sr/⁸⁶Sr signatures for Ayios Vasileios (n = 51) is drastically reduced compared to the bioavailable ⁸⁷Sr/⁸⁶Sr signatures recorded for its immediate surroundings, ranging only from 0.7082 to 0.7101. For Kalyvia (n = 3) and Voudeni (n = 99) the returned range in potentially local individual ⁸⁷Sr/⁸⁶Sr signatures is within 0.7080 to 0.7087 and, thus, almost identical to the reported range in bioavailable ⁸⁷Sr/⁸⁶Sr in their surrounding areas. The potentially local individuals from Kirrha (n = 29) returned a slightly more radiogenic ⁸⁷Sr/⁸⁶Sr range from 0.7084 to 0.7091. Finally, the most radiogenic range in Sr isotopes (0.7089–0.7105) was measured for the individuals excavated at Keros (n = 8) with most samples falling close to the single bioavailable baseline data point reported for its surrounding areas of 0.7093. The calculated site-specific Sr isotope baselines based on the human ⁸⁷Sr/⁸⁶Sr signatures of each site (Fig 2d, Table 1) generally defined narrower Sr isotope ranges as local for each site compared to the baselines of their surrounding areas. The human Sr isotope baselines of Kalyvia, Kirrha and Voudeni are still similar, with 0.7080 to 0.7089, 0.7084 to 0.7089 and 0.7081 to 0.7087, respectively. On the other hand, the human ⁸⁷Sr/⁸⁶Sr signatures of Ayios Vasileios and Keros define wider and generally more radiogenic baselines as local with 0.7084 to 0.7099 and 0.7084 to 0.7104, respectively.

## Discussion

### Baseline applicability

The calculated baselines for the surrounding areas of the investigated sites show a strong degree of overlap in their ⁸⁷Sr/⁸⁶Sr range (Fig 2b). The baselines of Apollo Maleatas, Kirrha, Kalyvia and Voudeni are almost identical in their range revealing that potential mobility between their surrounding areas would be undetectable utilizing Sr isotopes alone. Further, similar ranges in bioavailable ⁸⁷Sr/⁸⁶Sr have also been reported for many other Mediterranean regions, for example in Italy [45–47] or Cyprus [48], masking potential immigration from these regions.

The issue of undetectable migration using Sr isotopes is especially pronounced for Ayios Vasileios, whose surrounding area is characterized by a bioavailable Sr isotope baseline wide enough to encompass most of the bioavailable Sr isotope data reported for Europe thus far, e.g.,[14,17,18,46–49]. The wide range in bioavailable Sr isotope data reported around Ayios Vasileios has been attributed to the heterogenous surface geology of the southern Peloponnese [11,12]. While the archaeological site itself is located on geologically young, carbonate-rich sediments, which typically have ⁸⁷Sr/⁸⁶Sr signatures ~0.708 [50], it is flanked in the east by the Taygetos mountains with geologically older metamorphic outcrops resulting in bioavailable ⁸⁷Sr/⁸⁶Sr signatures as high as 0.723 [12]. Combining these two extreme end-members into a single baseline results in the wide ⁸⁷Sr/⁸⁶Sr baseline calculated for Ayios Vasileios surrounding area, which is practically unusable for the identification of individual mobility. However, the comparatively narrow range of skeletal ⁸⁷Sr/⁸⁶Sr values observed among individuals from Ayios Vasileios indicates that they did not source Sr evenly from both geological end-members. Instead, their diet was dominated by the unradiogenic carbonate source. This pattern may reflect the substantially lower Sr concentrations in soils developed on metamorphic outcrops relative to carbonate-rich soils [12,51]. Alternatively, it may indicate that the inhabitants of Ayios Vasileios preferentially obtained crops and drinking water from carbonate-dominated areas because these were closer or more suitable for agriculture. In either case, this example illustrates that bioavailable Sr isotope baselines derived from geologically heterogeneous terrains may not accurately represent the food and water actually consumed by past populations and are therefore unsuitable for mobility studies.

The problem of a biased Sr-uptake is accounted for by the site-specific skeletal ⁸⁷Sr/⁸⁶Sr isotope baselines, as demonstrated by the significantly narrower local ⁸⁷Sr/⁸⁶Sr range calculated for Ayios Vasileios (Fig 2d). Nevertheless, the skeletal ⁸⁷Sr/⁸⁶Sr isotope baseline of Ayios Vasileios is still significantly wider than any of the other sites located on the Peloponnese. This could suggest a high degree of non-locals at the site, which were overlooked due to wide baseline range of the surrounding area. However, previously reported environmental Sr isotope data confirmed a wider range in ⁸⁷Sr/⁸⁶Sr occurring naturally in the soils, plants and water around Ayios Vasileios, likely due to an influx of more radiogenic Sr from the mountains, than in the northern Peloponnese [12]. This suggests that the wide range in skeletal Sr isotope signatures observed for Ayios Vasileios is most likely the result of its setting, rather than human-induced processes, such as mobility or farming practices.

The skeletal ⁸⁷Sr/⁸⁶Sr baselines calculated for the investigated archaeological sites slightly improve the overlap between the baselines of Kirrha and Voudeni, suggesting that their inhabitants did not source Sr from their surrounding area homogeneously. Further, this method enabled the calculation of a local baseline for the site of Keros, whose surrounding area has not yet been sufficiently characterised by bioavailable proxy data, with only a single bioavailable Sr isotope value of 0.7093 reported over 20 km away on the island of Naxos [13] (Fig 1). On the other hand, the example of Apollo Maleatas also demonstrates the shortcoming of using skeletal ⁸⁷Sr/⁸⁶Sr signatures of ancient individuals to define a site-specific baseline for their dietary Sr intake, as two of the investigated three individuals had already been identified as likely non-local using the Sr isotope baseline of the surrounding area revealing a heavily biased dataset. Therefore, establishing local Sr isotope baselines from skeletal remains is problematic at archaeological sites with high levels of immigration, especially when sample sizes are small. A recent study on Bronze Age mobility in Northern Germany illustrates this issue, as the baseline derived from individual ⁸⁷Sr/⁸⁶Sr data differed markedly from that obtained from environmental samples of the surrounding area, likely reflecting an unrepresentative sampling of the local elite [52]. Consequently, a combined approach—first assessing the Sr isotope composition of the surrounding environment before defining site-specific skeletal baselines—appears most suitable for minimizing potential biases.

Finally, considering the large overlap in bioavailable ⁸⁷Sr/⁸⁶Sr signatures recorded for Greece [11–13,15,19] as well as the large overlap in calculated baselines within this study, it is very likely that some amount of individual mobility cannot be detected using Sr isotopes alone. Hence, the number of non-local individuals presented and discussed below should be considered representative of the minimum number rather than the absolute number of non-locals.

### Identifying non-locals

**Fig 3:**
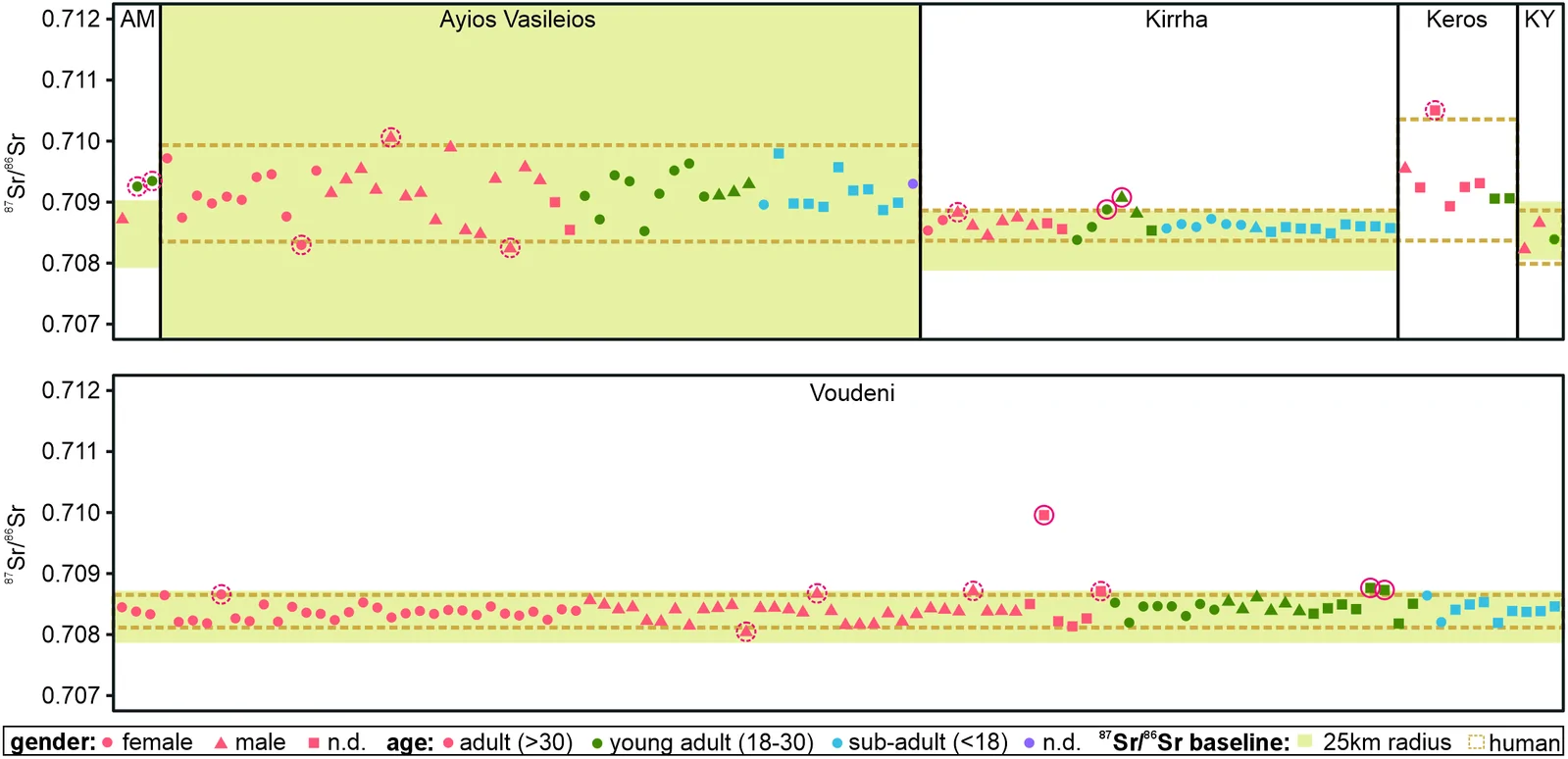
Skeletal ⁸⁷Sr/⁸⁶Sr isotope signatures of the investigated individuals from each site. The abbreviation KY stands for Kalyvia, n.d. for not determined and the ages of the different age groups are given in years. Individuals circled in red fall outside one (dotted line) or both (solid line) of their respective baselines.

Only 17 of the investigated 199 individuals have skeletal ⁸⁷Sr/⁸⁶Sr signatures outside one or both of the bioavailable Sr isotope baselines calculated for their burial site (Fig 3). This suggests that these individuals are non-locals and spent their childhood or parts thereof away from the investigated communities. However, it is notable that the offset between the individual ⁸⁷Sr/⁸⁶Sr signatures and their respective baselines varies drastically, with one individual from Voudeni (V066) falling 0.0012 above both its respective ⁸⁷Sr/⁸⁶Sr baseline ranges, while another (V008) falls only 8*10^−6^ above the human baseline and within the baseline of the surrounding area. Considering the above outlined uncertainty associated with defining local baselines, it is, therefore, important to remember that interpretations of foreign (or non-foreign) origin for the individuals presented here are based on the currently available baseline data and might change with improved data availability.

Multiple of the investigated individuals were already assessed as local or non-local based on Sr isotopes by a recent study [2]. Despite using different Sr isotope baselines the study generally identified the same individuals as non-local, supporting the suitability of our calculated baselines and, thus, the interpretations of their origin as foreign. The previously applied baselines were based on modern political boundaries, defining regional baselines based on available bioavailable Sr isotope data [11–13,15,17,19]. Using these baselines, eight potential non-locals were identified, five of which are confirmed by this study, including individual V008, who shows the smallest deviation from its respective baseline among all investigated individuals. None of the individuals previously interpreted as local by [2] fall outside the newly calculated site-specific baselines.

However, three individuals from Voudeni (V004, V071, V093) previously suggested as non-local [2], fall within the site-specific baselines defined in this study, indicating a likely local origin. This difference likely results from our use of site-specific rather than regional baselines and from defining baselines using double rather than single standard deviations. Given the low number of previously suggested non-locals at Voudeni (n = 8) relative to the total number of investigated individuals (n = 102), the Sr isotope baseline derived from the Voudeni individuals themselves likely provides the most accurate reflection of their dietary Sr intake. Hence, we propose that the ⁸⁷Sr/⁸⁶Sr signatures of V004, V071 and V093 should be considered within their local baseline. This does not exclude the possibility that they originated elsewhere, as all individuals could theoretically have come from a different locality with a similar Sr isotope signature.

### Mobility throughout the Bronze age

**Fig 4:**
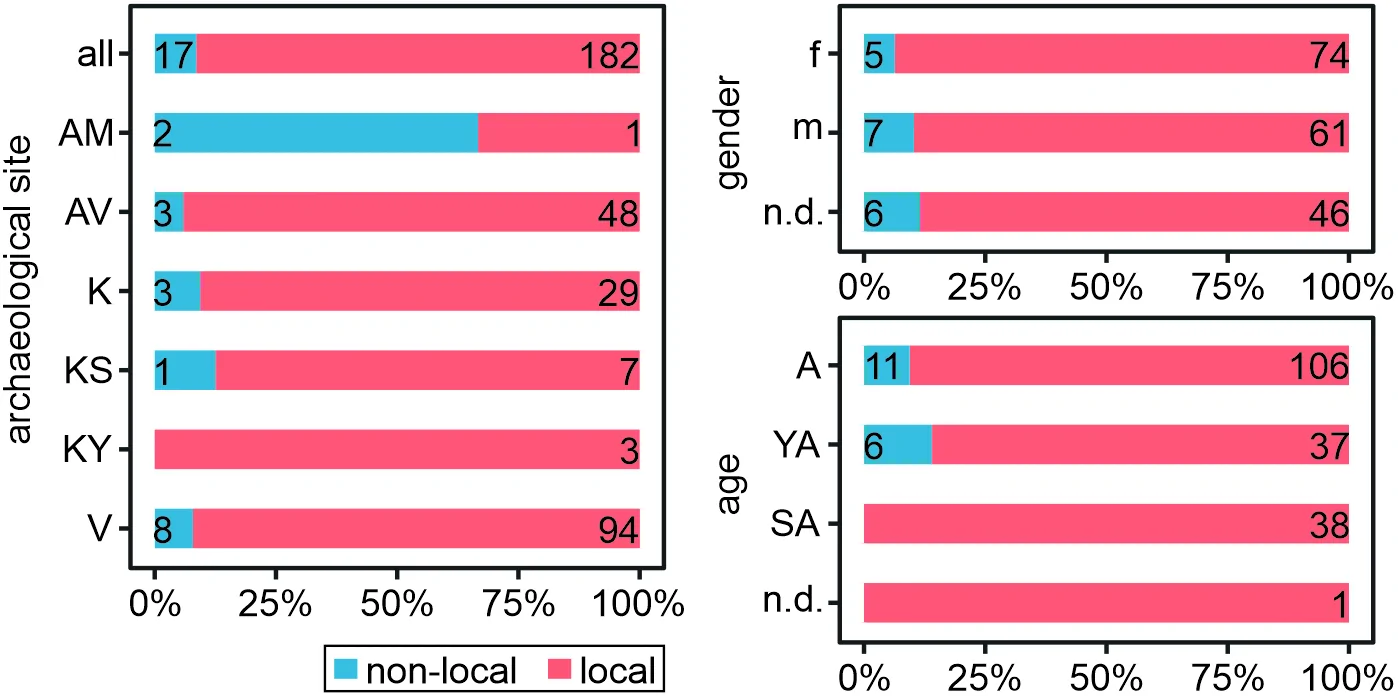
Percentage of non-local vs local individuals. Presented for the entire data set, each site, each gender and each age group. The numbers reflect the actual number of individuals in each group. The abbreviations are as follows: AM – Apollo Maleatas, AV – Ayios Vasileios, K – Kirrha, KS – Keros, KY – Kalyvia, V – Voudeni, f – female, m – male, A – adult, YA – young adult, SA – sub-adult, n.d. – not determined.

The investigated sites revealed that in total about 8.5% of the individuals buried there were not local to their respective sites (Fig 4). This suggests that the majority of individuals likely remained sedentary during the Bronze Age within this region of Greece despite the rapid cultural changes observed at this time, which are commonly assumed to be associated with extended human travel. This apparent disconnect could be due to methodological factors, such as an unrepresentative sample set or the overlapping Sr isotope baselines, or indicative of cultural factors, including mobility being limited to select individuals such as traders.

When looking at the individual sites, the percentage of non-locals varies drastically between 0 and 66.7%, however, most of that variability is due to Apollo Maleatas, Keros and Kalyvia being represented by a low number of individuals. As a result, our ability to determine the proportion of mobile individuals during the Neolithic and EBA is limited. Keros, which is represented by eight individuals, likely gives the best approximation with 12.5% non-locals, as one adult individual (KS03) returned a ⁸⁷Sr/⁸⁶Sr signature outside of the human Sr isotope baseline (Fig 3). At the same time, Keros is a very unique setting with no clear association to a particular settlement or formal cemetery, which complicates provenancing these individuals. Nevertheless, the vastly different ⁸⁷Sr/⁸⁶Sr signature of KS03 supports, that this individual grew up in a different region than the remaining individuals, identifying them as foreign to the community. Due to the lack of available baseline data within the surrounding area of the Keros burial site, it is currently not possible to determine whether the home community of those individuals was located close to their burial site. However, an origin in the South Aegean is supported for the seven individuals with a ⁸⁷Sr/⁸⁶Sr isotope signature compatible with the Keros human Sr isotope baseline based on a preliminary Sr baseline calculation for the region [11]. The ⁸⁷Sr/⁸⁶Sr signature of KS03, on the other hand, is too high for an origin within the South Aegean based on the current data availability, further supporting the identification of KS03 as non-local.

Other studies have applied Sr isotopes to investigate individual mobility in Neolithic and Early Bronze Age burials in central and southern Greece [15,16]. These studies generally found that fewer than 10% of individuals were non-local. In Attica, for example, the Tsepi (n = 75) and Loutsa (n = 13) cemeteries included only three individuals with non-local ⁸⁷Sr/⁸⁶Sr signatures, indicating that just 3.4% of the population originated elsewhere [15]. In the Cheliotomylos cemetery (n = 15) in Corinthia, four individuals were initially identified as falling outside the site-specific isotope baseline [15]. However, three of these had ⁸⁷Sr/⁸⁶Sr values very close to the upper baseline limit. Depending on whether only the clearly offset individual is considered non-local or all four are counted, the proportion of non-locals ranges from 6.7% to 26.7%, suggesting that the small sample size at Cheliotomylos limits the reliability of conclusions regarding mobility. On Crete, the Kephala Petras (n = 12) and Livari-Skiadi (n = 6) cemeteries included only one individual with a non-local Sr isotope signature, which is equivalent to only 5.6% of the population being foreign. These examples confirm that the calculated percentages of foreigners for Apollo Maleatas, Keros and Kalyvia are not representative for the general population dynamic of the Late Neolithic to EBA. When combining the Sr isotope results from all of these Neolithic to EBA cemeteries including Apollo Maleatas, Keros and Kalyvia (n = 135), only between eight and eleven non-locals could be identified in total, which is equivalent to a minimum mobility rate between 5.9% and 8.2% of the population during this time period in central and southern Greece.

The MBA to LBA settlements at Kirrha and Ayios Vasileios, are characterized by a non-local population of 9.4% and 5.9%, respectively (Fig 4). Hence, Ayios Vasileios shows a similar individual mobility rate as that observed for the Neolithic and the EBA communities, while Kirrha suggests a slight uptake in migration. It is important to note though, that Ayios Vasileios has one of the widest Sr isotope baselines defined in this study (Fig 2b , d), which could have resulted in a larger proportion of masked immigration from sites with overlapping baselines. Another thing that stands out about Ayios Vasileios is that the identified non-locals fall both below and above its site-specific baseline (Fig 3), revealing multi-directional immigration as these individuals had to come from areas with vastly different dietary Sr isotope compositions. A previous study on MBA to LBA mobility in southern Greece, investigated 30 individuals from four burial sites on Knossos for their Sr isotope composition [17]. The study considered all individuals to fall within their local baseline, suggesting a sedentary population. However, similar to Ayios Vasileios, the sites are characterised by a comparatively wide range in individual ⁸⁷Sr/⁸⁶Sr signatures and ⁸⁷Sr/⁸⁶Sr isotope baselines, suggesting that individual mobility could have been masked to some extent. Overall, in the complete Sr isotope data set of individuals from the MBA to LBA transition (n = 112) only 6 non-locals could be identified, supporting a minimum mobility rate of 5.4% of the population of southern and central Greece. This value is at the lower end of the mobility rate for Neolithic to EBA individuals, providing no evidence for an increased mobility towards the MBA to LBA transition based on Sr isotopes alone.

The LBA settlement, Voudeni, is characterised by 7.8% non-local individuals (Fig 4). Similar, to Ayios Vasileios, the non-locals from Voudeni are characterised by a variety of ⁸⁷Sr/⁸⁶Sr signatures, suggesting multi-directional immigration to the site (Fig 3). The comparatively low percentage of foreigners at Voudeni stands in contrast to a study on individual mobility of individuals from Mycenae (n = 11) in the Peloponnese, which suggested a high presence of non-locals (≥27.3%) within the shaft graves of Grave Circle A [14]. However, similar to Keros or Cheliotomylos, the number of investigated individuals is quite low, rendering it difficult to make meaningful interpretations. Further, the authors noted, that their defined Sr isotope baseline was based on a limited number of samples, potentially underrepresenting the local bioavailable Sr isotope composition, and, thus, considered only three individuals as certain non-locals [14]. Finally, differences in the chronological context, combined with the exceptional nature of Grave Circle A in Mycenae, complicate direct comparison. Combined the LBA sites reveal that out of all the individuals (n = 113) only eleven appear foreign to their settlement, which is equivalent to a minimum mobility rate of 9.8% for LBA individuals. This is slightly elevated compared to the earlier investigated time periods, but still quite low compared to other regions of Europe during the Bronze age, such as Italy [46,53] or Denmark [54]. Part of this difference could be due to the strongly overlapping baselines within Greece and with other Mediterranean countries [11–13,46–48,55]. However, the issue of overlapping baselines also exists for the above-mentioned contexts, hence these findings potentially indicate that the Bronze Age population of southern and central Greece might have been more sedentary compared to other regions during this period.

### Potential areas of origin

The identification of non-locals within an archaeological site always opens the question, where those individuals might have come from? Using Sr isotopes alone, this question cannot be readily answered as a non-local ⁸⁷Sr/⁸⁶Sr signature is generally compatible with multiple regions characterized by overlapping Sr isotope baselines. Hence, the area of origin of the individuals determined as foreign to their respective archaeological site cannot be determined with the available data, but only narrowed down to likely regions.

As discussed above, the non-local individuals of Ayios Vasileios and Voudeni fall above and below their respective baselines, emphasizing that they came from at least two different regions (Fig 3). Additionally, these non-local ⁸⁷Sr/⁸⁶Sr signatures range between 0.7086–0.7105 and 0.7080–07083, suggesting that the identified non-locals most likely came from more than three or four regions, thus, they provide evidence of multi-directional travel across or towards central and southern Greece during the Bronze Age.

Outside of Greece, bioavailable Sr isotope data compatible with either or both the low or high non-local ⁸⁷Sr/⁸⁶Sr signatures identified in this study has been reported for multiple countries, in particular around the Mediterranean, including Turkey [55,56], Cyprus [48], Italy [45–47], and Spain [51,57]. At the same time, all of the identified non-locals are also compatible with the available bioavailable Sr isotope data reported for modern day Greece [11], supporting the possibility of a strong local or inter-regional exchange rather than international travel. It is possible, probably even likely, that both types of migration occurred in Bronze Age Greece, but to what extent cannot be determined with the current data set.

In the following potential areas of origin within Greece are assessed. Hereby, it is notable that many of the identified non-locals still fall within or very close to the Sr isotope baseline (Δ⁸⁷Sr/⁸⁶Sr < 0.00006) defined for the surrounding area of their respective burial site (Fig 3). Hence, it cannot be ruled out that several of the potentially non-local individuals did not move from far away regions. Rather, they might have spent their youth in the surrounding area within a radius of approximately 25 km from their burial site. This would apply to all but one of the non-local individuals from Voudeni and Kirrha as well as all the non-local individuals from Ayios Vasileios. In the case of the latter, however, the calculated regional baseline is deemed unlikely to properly represent the actual bioavailable ⁸⁷Sr/⁸⁶Sr range of the area (see baseline discussion), as the large standard deviation and, thus, the baseline spread was caused by only two highly radiogenic ⁸⁷Sr/⁸⁶Sr values (Fig 2a). Thus, the low unradiogenic end of 0.702 of the calculated ⁸⁷Sr/⁸⁶Sr regional baseline of Ayios Vasileios is not actually supported by measured bioavailable ⁸⁷Sr/⁸⁶Sr data, but created statistically, and should not be applied. This suggests that two out of the three identified non-locals might not have come from within a 25 km radius. The geographically closest region compatible with the ⁸⁷Sr/⁸⁶Sr signature of these individuals (>0.7083), would be the northern Peloponnese, as suggested by the regional baselines of Voudeni, Kalyvia, and Apollo Maleatas (Fig 2b), as well as the local baseline of Mycenae [14]. This suggests a potential flow of people southwards within the Peloponnese.

Vice versa, the remaining non-locals from Voudeni (V66) and Kirrha (K14) as well as the identified foreigners from Apollo Maleatas (AM02 & AM03) and Keros (KS03) could have originated from the southern Peloponnese. Their non-local Sr isotope signatures fall substantially above their respective regional baseline (Δ⁸⁷Sr/⁸⁶Sr  < 0.00022), and are very similar to bioavailable Sr isotope data reported for the plains around the Taygetos mountains [12]. However, the southern Peloponnese is not the only region within modern-day Greece compatible with the Sr isotope composition of these non-locals, which could also have originated from parts of the North Aegean, as well as Central and West Macedonia [11,13,19,58]. Finally, it is important to remember that many regions around the Mediterranean, but also within modern-day Greece, are still underrepresented in terms of bioavailable ⁸⁷Sr/⁸⁶Sr baseline data, emphasizing that additional data is necessary to better constrain mobility pathways across Greece during the Bronze Age.

### The role of gender

A number of studies, in particular from prehistoric Europe, have highlighted a more or less strong gender bias in individual mobility patterns. For example, a high female mobility was observed for a final Neolithic to EBA settlement in southern Germany [53] as well as in a LBA community in northern Italy [46], suggesting that mobility was largely driven by patrilocal residential rules and female exogamy. On the other hand, a Sr study on a Bronze Age battlefield in northeast Germany unsurprisingly uncovered an overwhelming presence of male non-locals [59], revealing a male specific mobility bias.

Across the six Bronze Age cemeteries investigated in this study, 6.3% of the individuals identified as female (n = 84) and 10.3% of the individuals identified as male (n = 75) are characterized by a non-local ⁸⁷Sr/⁸⁶Sr signature for their respective burial site (Fig 4). This suggests a higher proportion of male mobility in central and southern Greece during the Bronze Age. However, it is also important to note that for 58 individuals the sex could not be determined, six of which were non-local. Depending on the gender of these foreigners the proportion of female to male mobility could change to a more gender-neutral trend.

When looking at the individual cemeteries no strong gender-bias can be observed as most cemeteries include non-locals of both gender and generally display a difference of only one individual between the non-local males and females (S1 Table, Fig 3). Voudeni is the only site that featured two more male non-locals than female, but also four foreigners of undetermined sex rendering its potential male driven mobility bias rather tentative. On the other hand, Apollo Maleatas revealed an indication of female driven mobility as the two non-locals are female, while the individual with a ⁸⁷Sr/⁸⁶Sr signature compatible with the local Sr isotope baseline is male. However, considering the low number of individuals no representative assessment of a gender bias can be made. Similarly, a female driven mobility was suggested for Mycenae, as the only two certainly female individuals were identified as potential non-locals [14]. However, the limited sample number and a lacking sex determination of the third non-local individual, also renders this interpretation tentative.

Other Bronze Age mobility studies from central and southern Greece have not yet assessed the role of gender for individual mobility [15–17]. The only other investigated cemetery with more than one identified non-local and available sex data is Tsepi in Attica, which identified two female and one probably male individual as foreign to the site [15], again, revealing travel for both genders. Hence, based on the currently available data, Bronze Age individual mobility does not appear to be driven by a specific gender-bias or one cultural practice.

### The role of age

A strength of Sr isotopes as a tracer of prehistoric mobility is the ability to create a timeline of when individuals moved in their lifetime by analysing multiple components of said individuals remains [60,61]. As such, it was possible to determine that a non-local young adult from Tsepi in Attica came there as an infant (before age of 3), while the non-local adult woman from Cheliotomylos in Corinthia moved there as an adult [15]. This was accomplished by analysing the first, second and third molar of these individuals, which form at different life stages, thus creating a timeline. As only one skeletal sample was analysed per individual in this study, no timeline for a specific individual could be created. However, as it was possible to assign a rough age range for most individuals based on osteological criteria (Fig 3, S1 Table), we can assess the proportion of non-locals of each age group to get an idea at what age individuals were most likely to travel.

Unlike the gender, the different age groups seem to reveal a mobility bias. None of the sub-adults (< 18 years of age) from the cemeteries investigated in this study appeared non-local to their respective site (Fig 3,4), suggesting that sub-adults were largely sedentary during the Bronze Age. This is not necessarily surprising, as children are commonly living with and reliant on their parents, thus they would likely only move if their parents moved. The group of the young adults (between 18 and 30 years of age) is marked by the highest proportion of non-local individuals with 14.0%, while only 9.4% of the adults (>30 years of age) were foreign. This suggests that Bronze Age individuals were most likely to move and settle away from their site of origin as young adults. It is important to note that the calculated percentages of non-locals within each age group should not be taken at face value, as the age at which the analysed adults moved to the site where they were buried, has not been established in all cases. Similarly, some of the non-local young adults could have already migrated during childhood, making it plausible that the mobility rate among sub-adults was higher than the suggested 0%, while the 9.4% mobility rate of adults should be considered the absolute maximum. This problem is illustrated by a non-local young adult from Tsepi [15], who was revealed to have moved before the age of three, as all three of their molars were analyzed for Sr. This example outlines the shortcomings of the Sr isotope method as applied in this study, as the Tsepi non-local could only be identified as foreign due to the analysis of their 1^st^ molar. Had the authors analyzed only the 2^nd^ or 3^rd^ molar, this young adult would have been considered as compatible with their local baseline. Hence, depending on which skeletal part was analyzed for the different individuals in this study, it is possible that individuals that moved very early in their childhood could not be identified as non-local as, e.g., the 3^rd^ molar does not form until an individual’s teenage years. Together with the issue of overlapping Sr isotope baselines within Greece, this emphasizes that the presented number of non-local individuals reported per site within this study, should be considered representative of the minimum amount of mobility.

Despite the limitations, this study suggests that young adults appear to be the most mobile group, suggesting that individuals are most likely to move between the ages of 18 and 30, and that there probably was a consistent individual mobility rather than the resettling of entire families, moving with their children. However, more mobility studies utilizing multiple skeletal samples from one and the same individuals are needed to create a more robust understanding of the role of age on Bronze age mobility in central and southern Greece.

### Mobility, kinship and status

While Sr isotopes are a powerful tool for exploring an individual’s mobility history, they are unsuitable to understand it in relation to the status or the family history of the analyzed individuals. Thus, Sr isotope data is commonly presented in combination with archaeological evidence, e.g.,[46,52,53] and increasingly also with aDNA data, e.g.,[2,62,63]. In the following we highlight available archaeological and aDNA evidence of identified non-locals to provide additional context on their mobility history.

Multiple studies have identified mobility among Bronze Age individuals in richly furbished or high status graves, e.g.,[46,52]. Within this study, three of the identified non-locals came from outstanding burials, which likely signify the individual’s high status. Two of these elite foreigners were unearthed at Voudeni (V70 & V66) from the same tomb (T75) [34], which is one of the two richest tombs of the cemetery, while the other highly ranked individual comes from Kirrha (K13) [64]. As K13 belongs to a period for which there is no clear evidence of settlement occupation at the site, even if a small and spatially restricted habitation may still have existed, her non-local origin in itself is not unexpected. More striking is the fact that she does not seem to have come from the newly established or more substantial communities in the immediate surroundings [65,66], nearby Krisa being the most obvious candidate, but rather from a more distant area. This further reinforces the singular character of her burial. Overall, the three outstanding burials from Voudeni and Kirrha constitute 17.7% of the non-locals identified within this study. While this is likely a higher proportion than that of high-status individuals in the overall population of these settlements, the obtained results still show that mobility was not constrained to the elite population.

Besides archaeological investigations, a recent aDNA study revealed that some of the identified non-locals in this study had relatives buried in the same cemetery [2]. Combining this knowledge with the Sr data presented here offers the unique opportunity to better understand the family dynamics of these individuals. Two of the individuals buried at Apollo Maleatas (AM01 & AM03), were identified as first-degree relatives and interpreted as a father (AM01)-daughter (AM03) duo [2]. Interestingly, the daughter was identified as non-local to the baseline of the surrounding area of Apollo Maleatas, while her father’s ⁸⁷Sr/⁸⁶Sr signature is compatible with it (S1 Table). There are multiple possibilities to explain this: 1) The father could have originated from a foreign region with overlapping baseline and have had his daughter while being mobile in a region with a more radiogenic baseline before eventually being buried in Apollo Maleatas; 2) He could have originated from Apollo Maleatas and left the area temporarily after the formation of his 3^rd^ molar, during which he had his daughter in a region with a more radiogenic baseline; 3) He could have been local to Apollo Maleatas throughout his lifetime and the foreign ⁸⁷Sr/⁸⁶Sr signature of his daughter reflects her mother’s (or another guardian’s) mobility.

Another set of first-degree relatives was discovered at Kirrha (K03 & K14) [2]. These individuals could either be father and son or two brothers. While both individuals were identified as non-local, they recorded different ⁸⁷Sr/⁸⁶Sr signatures, with K14 (⁸⁷Sr/⁸⁶Sr = 0.70907) falling outside of both baselines calculated for Kirrha and K03 (⁸⁷Sr/⁸⁶Sr = 0.70883) only barely outside the baseline calculated for the surrounding area (Δ⁸⁷Sr/⁸⁶Sr = 2*10^−7^). For individual K14 a 1^st^ molar was analysed, which starts forming already in utero, while canine teeth, which was analysed for K03, form later in life. This suggests that the measured Sr isotope signature of K14 likely reflects the childhood origin, while the signature of K03 could also be a transitional Sr isotope composition, if the individual migrated during childhood. Considering the close kinship of both individuals it is possible, if not likely, that both migrated to Kirrha together, with K14 recording the ⁸⁷Sr/⁸⁶Sr signature of their area of origin and K03 a transitional signal. Hence, if the individuals are a father-son duo, then K14 would have most likely been the father based on the ⁸⁷Sr/⁸⁶Sr signatures alone. However, ^14^C dating [2] and the fact that K03 is the secondary burial of a mature adult male placed in a pit next to the cist primary burial of the young male K14 suggests otherwise [33,64], indicating that K03 (who was older at death and also died earlier than K14) was the father or that the individuals might have been brothers. Sr analysis of additional tooth samples from both individuals might be able to further constrain their migratory pathway and help resolve the question of their kinship in the future.

## Conclusion

This study utilized Sr isotopes to investigate the childhood origin and mobility of Bronze Age individuals excavated from six different archaeological sites in central and southern Greece to better understand the potential role of migration in shaping changes in the local culture during this period. The results can be summarized as follows:

1. Choosing a baseline approach combining a baseline for the surrounding area of each site based on published data and a site-specific human baseline helped reduce baseline overlap as well as biases introduced by a small number of individuals, potential non-locals or extreme baseline values.
2. Of the 199 investigated individuals only 17 have ⁸⁷Sr/⁸⁶Sr signatures outside either one or both of the Sr isotope baselines defined for their respective burial site, suggesting these individuals originated elsewhere.
3. The percentage of non-locals identified for the Neolithic to EBA, the MBA to LBA and the LBA periods, were consistently between 5% and 10%, suggesting only a marginal increase towards the LBA. This suggests a constant flow of people to southern and central Greece throughout the Bronze Age, which seems lower than the rate obtained from other studies on prehistoric European communities.
4. The non-local individuals display a range in ⁸⁷Sr/⁸⁶Sr signatures suggesting multi-directional travel within southern and central Greece. While their non-local Sr isotope signatures are compatible with other Mediterranean countries, such as Italy or Cyprus, all of the non-local individuals could also have originated from another part of modern-day Greece.
5. The non-locals included individuals of both genders, with a slightly higher percentage of foreign males. However, when looking at the sites individually no clear gender-biased mobility driver could be identified suggesting that more than one cultural practice determined individual mobility.
6. The highest proportion of non-locals was found among the young adults, while sub-adults appear to be sedentary, suggesting that individual mobility, rather than the resettling of entire families, was predominant. However, as only one skeletal sample was analysed per individual, providing no mobility timelines, these findings should be considered tentative.
7. The archaeological evidence revealed that while some of the non-locals belonged to high ranking social groups, many of the non-locals were buried according to spread burial customs, suggesting that wealth was likely only one factor driving mobility.

## Supporting information

S1 Table Overview of individuals investigated in this study. Archive ID, gender, age and skeletal Sr isotope signature of the individuals from Apollo Maleatas, Ayios Vasileios, Kalyvia, Keros, Kirrha and Voudeni.
(XLSX)

## References

[1] Maran J, Wright JC. The rise of the Mycenaean culture, palatial administration and its collapse. A companion to the archaeology of early Greece and the Mediterranean. John Wiley & Sons, Ltd. 2019. p. 97–132. doi: 10.1002/9781118769966.ch5

[2] Yediay FE, Kroonen G, Sabatini S, Frei KM, Frank AB, Pinotti T. Ancient genomics support deep divergence between Eastern and Western Mediterranean Indo-European languages. bioRxiv. 2024. 2024.12.02.626332. doi: 10.1101/2024.12.02.626332

[3] Clemente F, Unterländer M, Dolgova O, Amorim CEG, Coroado-Santos F, Neuenschwander S, et al. The genomic history of the Aegean palatial civilizations. Cell. 2021;184(10):2565-2586.e21. doi: 10.1016/j.cell.2021.03.039 33930288 PMC8127963

[4] Lazaridis I, Mittnik A, Patterson N, Mallick S, Rohland N, Pfrengle S, et al. Genetic origins of the Minoans and Mycenaeans. Nature. 2017;548(7666):214–8. doi: 10.1038/nature23310 28783727 PMC5565772

[5] Skourtanioti E, Ringbauer H, Gnecchi Ruscone GA, Bianco RA, Burri M, Freund C, et al. Ancient DNA reveals admixture history and endogamy in the prehistoric Aegean. Nat Ecol Evol. 2023;7(2):290–303. doi: 10.1038/s41559-022-01952-3 36646948 PMC9911347

[6] Kotsonas A, Mokrišová J. Mobility, migration, and colonization. A companion to the archaeology of early Greece and the Mediterranean. John Wiley & Sons, Ltd. 2019. p. 217–46. doi: 10.1002/9781118769966.ch9

[7] Michael DE, Fibiger L, Moutafi I, Šlaus M, Katsimicha M, Novak M. Exploring connectivity in Late Bronze Age/Early Iron Age Greece and the Balkans using cranial non-metric analysis. Archaeological and Anthropological Sciences. 2023;15(11):165. doi: 10.1007/s12520-023-01862-w

[8] Montgomery J. Passports from the past: Investigating human dispersals using strontium isotope analysis of tooth enamel. Ann Hum Biol. 2010;37(3):325–46. doi: 10.3109/03014461003649297 20367186

[9] Alexander Bentley R. Strontium Isotopes from the Earth to the Archaeological Skeleton: A Review. J Archaeol Method Theory. 2006;13(3):135–87. doi: 10.1007/s10816-006-9009-x

[10] Flockhart DTT, Kyser TK, Chipley D, Miller NG, Norris DR. Experimental evidence shows no fractionation of strontium isotopes ((87)Sr/(86)Sr) among soil, plants, and herbivores: implications for tracking wildlife and forensic science. Isotopes Environ Health Stud. 2015;51(3):372–81. doi: 10.1080/10256016.2015.1021345 25789981

[11] Frank AB, Frei R, Moutafi I, Voutsaki S, Orgeolet R, Kristiansen K, et al. The geographic distribution of bioavailable strontium isotopes in Greece - A base for provenance studies in archaeology. Sci Total Environ. 2021;791:148156. doi: 10.1016/j.scitotenv.2021.148156 34126477

[12] Frank AB, Frei R, Triantaphyllou M, Vassilakis E, Kristiansen K, Frei KM. Isotopic range of bioavailable strontium on the Peloponnese peninsula, Greece: A multi-proxy approach. Science of The Total Environment. 2021;774:145181. doi: 10.1016/j.scitotenv.2021.145181

[13] Nafplioti A. Tracing population mobility in the Aegean using isotope geochemistry: a first map of local biologically available 87Sr/86Sr signatures. J Archaeol Sci. 2011;38(7). doi: 10.1016/j.jas.2011.02.021

[14] Nafplioti A. Mycenae Revisited Part 2. Exploring the Local versus Non-local Geographical Origin of the Individuals from Grave Circle A: Evidence from Strontium Isotope Ratio (87Sr/86Sr) Analysis. Annu Br Sch Athens. 2009;104:279–91. doi: 10.1017/s0068245400000253

[15] Prevedorou EA. The role of kin relations and residential mobility during the transition from final Neolithic to early Bronze Age in Attica, Greece. Phoenix, AZ: Arizona State University. 2015.

[16] Triantaphyllou S, Nikita E, Kador T. Exploring Mobility Patterns And Biological Affinities In The Southern Aegean: First Insights From Early Bronze Age Eastern Crete. Annu Br Sch Athens. 2015;110:3–25. doi: 10.1017/s0068245415000064

[17] Nafplioti A. Mycenaean political domination of Knossos following the Late Minoan IB destructions on Crete: negative evidence from strontium isotope ratio analysis (87Sr/86Sr). J Archaeol Sci. 2008;35(8):2307–17. doi: 10.1016/j.jas.2008.03.006

[18] Richards M, Harvati K, Grimes V, Smith C, Smith T, Hublin JJ. Strontium isotope evidence of Neanderthal mobility at the site of Lakonis, Greece using laser-ablation PIMMS. J Archaeol Sci. 2008;35(5):1251–6. doi: 10.1016/j.jas.2007.08.018

[19] Hoogewerff JA, Reimann C, Ueckermann H, Frei R, Frei KM, van Aswegen T, et al. Bioavailable 87Sr/86Sr in European soils: A baseline for provenancing studies. Sci Total Environ. 2019;672:1033–44. doi: 10.1016/j.scitotenv.2019.03.387 30999220

[20] GADM. GADM maps and data. https://gadm.org/ Accessed 2020 August 6.

[21] Theodorou-Mavrommatidi AI. An early Helladic settlement in the Apollon Maleatas site at Epidauros. Die Ägäische Frühzeit, 2. Serie: Forschungsbericht 1975-2000: 2. Band: Teil 2: Die Frühbronzezeit in Griechenland mit Ausnahme von Kreta. Wien: Verlag der Österreichischen Akademie der Wissenschften. 2004. p. 1167–82.

[22] Theodorou-Mavrommatidi AA. Defining ritual action. A Middle Helladic pit at the site of Apollon Maleatas in Epidauros. Mesohelladika. The Greek Mainland in the Middle Bronze Age. Leuven: Peeters Publishers. 2010. p. 521–33.

[23] Voutsaki S, Moutafi I, Hachtmann V. The North Cemetery Ayios Vasileios. Austerity and differentiation in the Early Mycenaean period. Middle and Late Helladic Laconia: Competing principalities?. Sidestone Press. 2022.

[24] Erdil P, Dee MW, Kuitems M, Moutafi I, Hachtmann V, Vika E, et al. The absolute chronology of the North Cemetery at Ayios Vasileios, Laconia. J Archaeol Sci Rep. 2024;55:104457. doi: 10.1016/j.jasrep.2024.104457

[25] Moutafi I, Voutsaki S. Commingled burials and shifting notions of the self at the onset of the Mycenaean era (1700–1500 BCE): The case of the Ayios Vasilios North Cemetery, Laconia. J Archaeol Sci Rep. 2016;10:780–90. doi: 10.1016/j.jasrep.2016.05.037

[26] Rambach J. The early Helladic I cemetery at Kalyvia in ancient Elis. Communities in transition, the circum-Aegean area during the 5th and 4th millennia BC. Oxford: Oxbow Books. 2018. p. 341–9.

[27] Renfrew C, Boyd MJ, Athanasoulis D, Brodie N, Carter T, Dellaporta K, et al. The sanctuary at Keros in the Aegean Early Bronze Age: from centre of congregation to centre of power. JGA. 2022;7:1–36. doi: 10.32028/jga.v7i.1709

[28] Renfrew C, Boyd M, Ramsey CB. The oldest maritime sanctuary? Dating the sanctuary at Keros and the Cycladic Early Bronze Age. Antiquity. 2012;86(331):144–60. doi: 10.1017/S0003598X00062517

[29] Renfrew C. The Sanctuary at Keros: Questions of materiality and monumentality. Proc Br Acad. 2013;1:187–212. doi: 10.5871/bacad/9780197265666.003.0008

[30] Renfrew C, Moutafi I. Area A. The Sanctuary on Keros and the Origins of Aegean Ritual Practice: The Excavations of 2006-2008. Cambridge: Cambridge University Press. 2015. p. 457–82.

[31] Moutafi I. The human remains from Area A. The sanctuary on Keros and the origins of Aegean ritual practice: The excavations of 2006-2008. Cambridge: Cambridge University Press. 2015. p. 483–505.

[32] Dor L, Jannoray J, Van Effenterre H, Van Effenterre M. Kirrha. Etude de préhistoire phocidienne. Paris: De Boccard. 1960.

[33] Lagia A, Moutafi I, Orgeolet R, Skorda D, Zurbach J. Revisiting the tomb: Mortuary practices in habitation areas in the transition to the late Bronze Age at Kirrha, Phocis. Staging death: Funerary performance, architecture and landscape in the Aegean. Berlin: De Gruyter. 2016. p. 206–32.

[34] Vounteni K, Lazaros I. An important mycenaean centre of achaea – volume a. Athen: A. 2024.

[35] Moutafi I. Towards a social bioarchaeology of the Mycenaean period: A biocultural analysis of human remains from the Voudeni cemetery, Achaea, Greece. Oxbow Books. 2021. doi: 10.2307/j.ctv13nb971

[36] Harvig L, Frei KM, Price TD, Lynnerup N. Strontium isotope signals in cremated petrous portions as indicator for childhood origin. PLoS One. 2014;9(7):e101603. doi: 10.1371/journal.pone.0101603 25010496 PMC4091946

[37] Veselka B, Locher H, de Groot JCMJ, Davies GR, Snoeck C, Kootker LM. Strontium isotope ratios related to childhood mobility: Revisiting sampling strategies of the calcined human pars petrosa ossis temporalis. Rapid Commun Mass Spectrom. 2021;35(7):e9038. doi: 10.1002/rcm.9038 33370492

[38] Buikstra JE. Standards for data collection from human skeletal remains. In: Ubelaker DH. Fayetteville, Arkansas: Arkansas Archeological Survey. 1994.

[39] AlQahtani SJ, Hector MP, Liversidge HM. Brief communication: The London atlas of human tooth development and eruption. Am J Phys Anthropol. 2010;142(3):481–90. doi: 10.1002/ajpa.21258 20310064

[40] Liversidge HM, Molleson T. Variation in crown and root formation and eruption of human deciduous teeth. Am J Phys Anthropol. 2004;123(2):172–80. doi: 10.1002/ajpa.10318 14730650

[41] Scheuer L, Black S. Developmental juvenile osteology. London. 2000.

[42] Frei KM, Frei R. The geographic distribution of strontium isotopes in Danish surface waters – a base for provenance studies in archaeology, hydrology and agriculture. Appl Geochem. 2011;26(3):326–40. doi: 10.1016/j.apgeochem.2010.12.006

[43] Thirlwall MF. Long-term reproducibility of multicollector Sr and Nd isotope ratio analysis. Chem Geol. 1991;94(2):85–104. doi: 10.1016/S0009-2541(10)80021-X

[44] Frei KM, Beck MR, Pantmann P, Møller NA, Søvsø M, Frei R. Bronze Age non-elite mobility in Denmark examined through a new human-based bioavailable strontium isotope range. PLoS One. 2026;21(2):e0341434. doi: 10.1371/journal.pone.0341434 41650112 PMC12880727

[45] Ladegaard-Pedersen P, Frei R, Frank AB, Saracino M, Zorzin R, Martinelli N. Constraining a bioavailable strontium isotope baseline for the Lake Garda region, Northern Italy: A multi-proxy approach. J Archaeol Sci Rep. 2022;41:103339. doi: 10.1016/j.jasrep.2022.103339

[46] Cavazzuti C, Skeates R, Millard AR, Nowell G, Peterkin J, Bernabò Brea M, et al. Flows of people in villages and large centres in Bronze Age Italy through strontium and oxygen isotopes. PLoS One. 2019;14(1):e0209693. doi: 10.1371/journal.pone.0209693 30625174 PMC6326466

[47] Lugli F, Cipriani A, Bruno L, Ronchetti F, Cavazzuti C, Benazzi S. A strontium isoscape of Italy for provenance studies. Chem Geol. 2022. doi: 10.1016/j.chemgeo.2021.120624

[48] Ladegaard-Pedersen P, Achilleos M, Dörflinger G, Frei R, Kristiansen K, Frei KM. A strontium isotope baseline of Cyprus. Assessing the use of soil leachates, plants, groundwater and surface water as proxies for the local range of bioavailable strontium isotope composition. Sci Total Environ. 2020;708:134714. doi: 10.1016/j.scitotenv.2019.134714 31787293

[49] Voerkelius S, Lorenz GD, Rummel S, Quétel CR, Heiss G, Baxter M, et al. Strontium isotopic signatures of natural mineral waters, the reference to a simple geological map and its potential for authentication of food. Food Chemistry. 2010;118(4):933–40. doi: 10.1016/j.foodchem.2009.04.125

[50] McArthur JM, Howarth RJ, Shields GA, Zhou Y. Strontium Isotope Stratigraphy. Geologic Time Scale 2020. Elsevier. 2020. p. 211–38. doi: 10.1016/b978-0-12-824360-2.00007-3

[51] Frank AB, Frei R, Kristiansen K, Frei KM. A multi-proxy, bioavailable strontium isotope baseline for southern Almería, Spain: Using modern environmental samples to constrain the isotopic range of bioavailable strontium. Appl Geochem. 2022;144:105405. doi: 10.1016/j.apgeochem.2022.105405

[52] Frank AB, May J, Sabatini S, Schopper F, Frei R, Kaul F, et al. A Late Bronze Age foreign elite? Investigating mobility patterns at Seddin, Germany. PLoS One. 2025;20(9):e0330390. doi: 10.1371/journal.pone.0330390 40929027 PMC12422465

[53] Knipper C, Mittnik A, Massy K, Kociumaka C, Kucukkalipci I, Maus M, et al. Female exogamy and gene pool diversification at the transition from the Final Neolithic to the Early Bronze Age in central Europe. Proceedings of the National Academy of Sciences. 2017;114(38):10083–8. doi: 10.1073/pnas.1706355114 PMC5617280 28874531

[54] Frei KM, Bergerbrant S, Sjögren K-G, Jørkov ML, Lynnerup N, Harvig L, et al. Mapping human mobility during the third and second millennia BC in present-day Denmark. PLoS One. 2019;14(8):e0219850. doi: 10.1371/journal.pone.0219850 31433798 PMC6703675

[55] Wong M, Brandt JR, Ahrens S, Jaouen K, Bjørnstad G, Naumann E. Pursuing pilgrims: isotopic investigations of Roman and Byzantine mobility at Hierapolis, Turkey. J Archaeol Sci Rep. 2018;17:520–8. doi: 10.1016/j.jasrep.2017.12.005

[56] Yazıcıoğlu GB, Meiggs DC, Davis M, Pilaar Birch SE. Emerging strontium isoscapes of Anatolia (Türkiye): new datasets and perspectives in bioavailable 87Sr/86Sr baseline studies. Front Environ Archaeol. 2025;4. doi: 10.3389/fearc.2025.1520345

[57] Valenzuela-Lamas S, Orengo HA, Bosch D, Pellegrini M, Halstead P, Nieto-Espinet A, et al. Shipping amphorae and shipping sheep? Livestock mobility in the north-east Iberian peninsula during the Iron Age based on strontium isotopic analyses of sheep and goat tooth enamel. PLoS One. 2018;13(10):e0205283. doi: 10.1371/journal.pone.0205283 30379834 PMC6209160

[58] Whelton HL, Lewis J, Halstead P, Isaakidou V, Triantaphyllou S, Tzevelekidi V. Strontium isotope evidence for human mobility in the Neolithic of northern Greece. J Archaeol Sci Rep. 2018;20:768–74. doi: 10.1016/j.jasrep.2018.06.020

[59] Price TD, Frei R, Brinker U, Lidke G, Terberger T, Frei KM, et al. Multi-isotope proveniencing of human remains from a Bronze Age battlefield in the Tollense Valley in northeast Germany. Archaeol Anthropol Sci. 2019;11(1):33–49. doi: 10.1007/s12520-017-0529-y

[60] Frei KM, Mannering U, Price TD, Iversen RB. Strontium isotope investigations of the Haraldskær Woman – a complex record of various tissues. ArcheoSciences Rev Archéom. 2015;39:93–101. doi: 10.4000/archeosciences.4407

[61] Frei KM, Mannering U, Kristiansen K, Allentoft ME, Wilson AS, Skals I. Tracing the dynamic life story of a Bronze Age female. Sci Rep. 2015;5:10431. doi: 10.1038/srep10431 PMC4440039 25994525

[62] Richards M, Smith C, Nehlich O, Grimes V, Weston D, Mittnik A, et al. Finding Mycenaeans in Minoan Crete? Isotope and DNA analysis of human mobility in Bronze Age Crete. PLoS One. 2022;17(8):e0272144. doi: 10.1371/journal.pone.0272144 35947574 PMC9365174

[63] Haak W, Brandt G, Jong HNd, Meyer C, Ganslmeier R, Heyd V. Ancient DNA, strontium isotopes, and osteological analyses shed light on social and kinship organization of the Later Stone Age. Proceedings of the National Academy of Sciences. 2008;105(47):18226–31. doi: 10.1073/pnas.0807592105 PMC2587582 19015520

[64] Orgeolet R, Skorda D, Zurbach J, Barbarin L de, Bérard RM, Chevaux B, et al. Kirrha 2008‑2015 : un bilan d’étape. La fouille et les structures archéologiques. Bull Corresp Hell. 2017;141(1):141.1. doi: 10.4000/bch.519

[65] Effenterre HV, Jannoray J. Fouilles de Krisa. Bull Corresp Hell. 1938;62(1):110–48. doi: 10.3406/bch.1938.2698

[66] Müller S. Delphes et sa région à l’époque mycénienne. 1992. doi: 10.3406/bch.1992.4664

