# Supplementary material for: Exploring mobility in Bronze Age Greece: A Sr isotope perspective
Source: PLoS One. 2026 Sep 23;21(9):e0357576. doi: 10.1371/journal.pone.0357576 (PMC13600528; doi:10.1371/journal.pone.0357576)
Supplement: Supplementary file 1 [file pone.0357576.s003.docx]

Dear Editor,

Thank you for considering our manuscript titled ‘Exploring mobility in Bronze Age Greece: A Sr isotope perspective’ for publication in PlosONE. We’ve copied the outlined journal requirements and reviewer comments in black below and added how we addressed these in blue.

Journal Requirements:

When submitting your revision, we need you to address these additional requirements.

1. Please ensure that your manuscript meets PLOS ONE's style requirements, including those for file naming. The PLOS ONE style templates can be found at

https://journals.plos.org/plosone/s/file?id=wjVg/PLOSOne_formatting_sample_main_body.pdf and https://journals.plos.org/plosone/s/file?id=ba62/PLOSOne_formatting_sample_title_authors_affiliations.pdf

We’ve ensured that our article meets the journal requirements.

2. Please include a complete copy of PLOS’ questionnaire on inclusivity in global research in your revised manuscript. Our policy for research in this area aims to improve transparency in the reporting of research performed outside of researchers’ own country or community. The policy applies to researchers who have travelled to a different country to conduct research, research with Indigenous populations or their lands, and research on cultural artefacts. The questionnaire can also be requested at the journal’s discretion for any other submissions, even if these conditions are not met. Please find more information on the policy and a link to download a blank copy of the questionnaire here: https://journals.plos.org/plosone/s/best-practices-in-research-reporting. Please upload a completed version of your questionnaire as Supporting Information when you resubmit your manuscript.

We’ve filled out and included the questionnaire for our resubmission.

3. Please provide a complete Data Availability Statement in the submission form, ensuring you include all necessary access information or a reason for why you are unable to make your data freely accessible. If your research concerns only data provided within your submission, please write "All data are in the manuscript and/or supporting information files" as your Data Availability Statement.

All data are in the manuscript and/or supporting information files.

4. We note that the grant information you provided in the ‘Funding Information’ and ‘Financial Disclosure’ sections do not match.

When you resubmit, please ensure that you provide the correct grant numbers for the awards you received for your study in the ‘Funding Information’ section.

We’ve amended our financial disclosure to align it with the funding information:

The Sr isotope work of this study was made possible through the funding provided by the “Semper Ardens” research grant CF18-0005 from Carlsbergfondet (KMF; https://www.carlsbergfondet.dk/en) and the Jubileumsfond grant M16-0455 :1 from Sveriges Riksbanken (KK; https://www.rj.se/en/). The bioarchaeological study was supported by the European Union’s Marie Skłodowska-Curie Actions grant No. 747458 (IM; https://marie-sklodowska-curie-actions.ec.europa.eu/). The funders had no role in study design, data collection and analysis, decision to publish, or preparation of the manuscript.

5. Thank you for stating the following financial disclosure:

The Sr isotope work of this study was made possible through the funding provided by the “Semper Ardens” research grant CF18-0005 from Carlsbergfondet (KMF; https://www.carlsbergfondet.dk/en) and the Jubileumsfond grant M16-0455 :1 from Sveriges Riksbanken (KK; https://www.rj.se/en/). The bioarchaeological study was supported by the European Union’s Marie Skłodowska-Curie Actions grant No. 747458 (IM; https://marie-sklodowska-curie-actions.ec.europa.eu/), and the Malcolm H. Wiener Laboratory for Archaeological Science Post-doctoral Research Fellowship (IM; https://www.ascsa.edu.gr/research/wiener-laboratory).

Please state what role the funders took in the study. If the funders had no role, please state: "The funders had no role in study design, data collection and analysis, decision to publish, or preparation of the manuscript."

If this statement is not correct you must amend it as needed.

Please include this amended Role of Funder statement in your cover letter; we will change the online submission form on your behalf.

We’ve amended our statement to include that the funders had no role in study design, data collection and analysis, decision to publish, or preparation of the manuscript (also see comment above).

6. We note that Figure 1 in your submission contain [map/satellite] images which may be copyrighted. All PLOS content is published under the Creative Commons Attribution License (CC BY 4.0), which means that the manuscript, images, and Supporting Information files will be freely available online, and any third party is permitted to access, download, copy, distribute, and use these materials in any way, even commercially, with proper attribution. For these reasons, we cannot publish previously copyrighted maps or satellite images created using proprietary data, such as Google software (Google Maps, Street View, and Earth). For more information, see our copyright guidelines: http://journals.plos.org/plosone/s/licenses-and-copyright.

We require you to either (1) present written permission from the copyright holder to publish these figures specifically under the CC BY 4.0 license, or (2) remove the figures from your submission:

a. You may seek permission from the original copyright holder of Figure 1 to publish the content specifically under the CC BY 4.0 license.

We recommend that you contact the original copyright holder with the Content Permission Form (http://journals.plos.org/plosone/s/file?id=7c09/content-permission-form.pdf) and the following text:

“I request permission for the open-access journal PLOS ONE to publish XXX under the Creative Commons Attribution License (CCAL) CC BY 4.0 (http://creativecommons.org/licenses/by/4.0/). Please be aware that this license allows unrestricted use and distribution, even commercially, by third parties. Please reply and provide explicit written permission to publish XXX under a CC BY license and complete the attached form.”

Please upload the completed Content Permission Form or other proof of granted permissions as an "Other" file with your submission.

In the figure caption of the copyrighted figure, please include the following text: “Reprinted from [ref] under a CC BY license, with permission from [name of publisher], original copyright [original copyright year].”

b. If you are unable to obtain permission from the original copyright holder to publish these figures under the CC BY 4.0 license or if the copyright holder’s requirements are incompatible with the CC BY 4.0 license, please either i) remove the figure or ii) supply a replacement figure that complies with the CC BY 4.0 license. Please check copyright information on all replacement figures and update the figure caption with source information. If applicable, please specify in the figure caption text when a figure is similar but not identical to the original image and is therefore for illustrative purposes only.

The following resources for replacing copyrighted map figures may be helpful:

USGS National Map Viewer (public domain): http://viewer.nationalmap.gov/viewer/

The Gateway to Astronaut Photography of Earth (public domain): http://eol.jsc.nasa.gov/sseop/clickmap/

Maps at the CIA (public domain): https://www.cia.gov/library/publications/the-world-factbook/index.html and https://www.cia.gov/library/publications/cia-maps-publications/index.html

NASA Earth Observatory (public domain): http://earthobservatory.nasa.gov/

Landsat: http://landsat.visibleearth.nasa.gov/

USGS EROS (Earth Resources Observatory and Science (EROS) Center) (public domain): http://eros.usgs.gov/#

Natural Earth (public domain): http://www.naturalearthdata.com/

The map was created from scratch with no copyrighted imagery and can thus be published under CC BY 4.0 without any citations.

7. Please include a copy of Table 3, which you refer to in your text on page 8, 9.

Sorry for this oversight, these citations were from an earlier draft and were, therefore, deleted.

8. We note that there is identifying data in the Supporting Information file <PlosONE_Greece_tablesS1_1.xlsx>. Due to the inclusion of these potentially identifying data, we have removed this file from your file inventory. Prior to sharing human research participant data, authors should consult with an ethics committee to ensure data are shared in accordance with participant consent and all applicable local laws.

Data sharing should never compromise participant privacy. It is therefore not appropriate to publicly share personally identifiable data on human research participants. The following are examples of data that should not be shared:

-Name, initials, physical address

-Ages more specific than whole numbers

-Internet protocol (IP) address

-Specific dates (birth dates, death dates, examination dates, etc.)

-Contact information such as phone number or email address

-Location data

-ID numbers that seem specific (long numbers, include initials, titled “Hospital ID”) rather than random (small numbers in numerical order)

Data that are not directly identifying may also be inappropriate to share, as in combination they can become identifying. For example, data collected from a small group of participants, vulnerable populations, or private groups should not be shared if they involve indirect identifiers (such as sex, ethnicity, location, etc.) that may risk the identification of study participants.

Additional guidance on preparing raw data for publication can be found in our Data Policy (https://journals.plos.org/plosone/s/data-availability#loc-human-research-participant-data-and-other-sensitive-data) and in the following article: http://www.bmj.com/content/340/bmj.c181.long.

Please remove or anonymize all personal information (<specific identifying information in file to be removed>), ensure that the data shared are in accordance with participant consent, and re-upload a fully anonymized data set. Please note that spreadsheet columns with personal information must be removed and not hidden as all hidden columns will appear in the published file.

There is no identifying data included in Table S1. The investigated individuals lived and died thousands of years ago and their identities have been lost to history. The included Archive IDs are the assigned IDs to the sampled teeth and bones (not full individuals) and are included to help future scientists interested in conducting further research to identify and find the samples in question. This was done to adhere to the FAIR principles.

9. If the reviewer comments include a recommendation to cite specific previously published works, please review and evaluate these publications to determine whether they are relevant and should be cited. There is no requirement to cite these works unless the editor has indicated otherwise.

The only suggested reference was a prehistoric Aegean DNA study, that was already included in the study. Thus, it is appropriate for the study and we added a sentence/citation expanding on the study’s findings.

10. Please review your reference list to ensure that it is complete and correct. If you have cited papers that have been retracted, please include the rationale for doing so in the manuscript text, or remove these references and replace them with relevant current references. Any changes to the reference list should be mentioned in the rebuttal letter that accompanies your revised manuscript. If you need to cite a retracted article, indicate the article’s retracted status in the References list and also include a citation and full reference for the retraction notice.

We could not find any issues with our reference list. We’ve added only two references to address the concerns of Reviewer 2 regarding the suitability of petrous bones (see comments below).

Comments to the Author

Reviewer #1: Based on a data base of samples of the skeletal remains of 199 individuals from six archaeological sites and due to a carefully designed mathodological approach, the article constitutes an important addition to our knowledge about applying the method of Sr-isotopy to gain insights into issues of mobility in Bronze Age Greece. The authors demonstrate that, if applied to human remains in Greece, the results of this method are far less equivocal than in other parts of the world, because, due to similar geological circumstances, the Sr-baselines of many Greek regions overlap to a certain degree. To counter these problems, the authors have refined their approach by combining a Sr-baseline based on the geological data available for the surroundings of a site with a site-specific human baseline. In this way, they are able to mitigate the problem of overlapping baselines which allows them to define a certain number of non-local people in the respective data sets of the investigated sites. Their calculation of percentages between 5 and 10% of non-locals throughout the Bronze Age should be regarded as a minimum, since, as the authors stress, there must be many non-locals that remain undetected due to the still existing significant overlap of Sr-baseline between various regions of Greece and even other parts of the Mediterranean. Important are also the conclusions that the identified non-locals were predominantly young adults and that these patterns of mobility do not seem to have been gender-specific.
In the general, the manuscript is carefully written, but I found a small number of spelling mistakes:
- p. 25 "Southern Germany" (not "Southern German");
- p. 25: "as well as a in a LBA community" (not: "as well as a in LBA community");
- p. 30: "only 17 have....signatures") (not: "only 17 have.....signature").

Thank you for the careful review. We’ve made the necessary adjustments.

Reviewer #2: With their article, Frank et al. fill a most important gap of bioarchaeological research in Greece, namely Strontium isotope data. The last years have seen an increasing number of other bioarchaeological datasets, especially in the field of archaeogenetics, but Strontium data have been scarce. The publication of 199 skeletal samples, therefore, presents an enormous step forward.

I think that the article could profit from some more precision through. This is particularly when the authors speak about the “Bronze Age”. When they describe the Bronze Age, e.g. “saw rapid economic and cultural changes” (l. 28) and that “Greece was a centre for economic and cultural innovation” (l. 48-49) (whereby I was asking myself, which cultural innovation the authors refer to…). Whereas this is true for some parts of the Bronze Age, this can definitely not be said for the whole period from 3000 to 1050 BC with all its non-linear developments, social transformations and regional specificities. During the Middle Bronze Age, Crete was indeed an economic and cultural center, whereas the Mainland was far from that etc. Here, I think the authors should avoid such overarching generalisations about THE “Greek Bronze Age” and be more specific in a spatio-temporal perspective when they refer to cultural topics.

We’ve amended the starting sentence to the Abstract (l.28) to: ‘The Mediterranean region saw significant socio-economic and cultural transformations during the Bronze Age, varying across regions and through time. Expanding exchange networks played an important role in these developments by connecting distant communities.’, to introduce the variability in the mentioned economic and cultural developments.

Further we extended our introduction (originally l. 48-59) to elaborate further. It now reads as follows:

‘During the Bronze Age, the region of modern-day Greece underwent substantial social, economic, and cultural transformations, differently expressed across regions and through time. In particular, the transition from the Middle to Late Bronze Age witnessed the formation of Mycenaean societies on the mainland, accompanied by increasing socio-political complexity, expanding exchange networks, and significant changes in settlement organisation and material culture. Following diverse local trajectories, these developments were crucial for the emergence of Mycenaean palatial societies and continued to shape communities during the palatial and post-palatial times of the Late Bronze Age. Archaeological evidence has revealed major changes to mortuary practices, domestic architecture and settlement organisation [1], while recent and ongoing genetic studies suggest an increasingly complex demographic history, including the arrival of populations carrying Steppe-related ancestry beginning around 2200 BC [2–5,5]. However, patterns of individual mobility within local communities remain poorly understood. Exchange of goods, ideas and cultural practices is well attested across the Mediterranean and the Aegean throughout the Bronze Age, although its intensity and character varied across regions and periods [6]; to what degree this was accompanied by migration of foreign individuals to local communities has not been yet thoroughly investigated. Indeed, archaeological evidence fails to determine whether goods and ideas were introduced by traders, which never entered the local community, or through individual migration and settlement to the local community. Although ancient DNA studies have recently revealed otherwise undetected evidence of past mobility by identifying Anatolian ancestry for prehistoric individuals from the Greek Island Euboea [5], bioarchaeological research on the question of human mobility during this period is still fairly limited [7].’

As it is stated in the materials chapter, the study included teeth and petrous bones for Sr analysis. Here, I think it would have been essential to further elaborate on the sampling strategy. What is the share of the different molars among the samples (M1, M2, M3)? The authors just state that M1 and M2 were preferred. Why did they select petrous bones instead of teeth in some cases and what was the sampling strategy behind? The reviewer is aware of the fragmentary preservation of skeletal material, especially in the Bronze Age collective burials of the Aegean. However, I think it is necessary to elaborate on the inherent challenges of sampling and comparing different types of samples as well as different molars and how this influences the results. Moreover, some scholars have argued that the petrous portion might take up Sr isotopes from the soil post-mortem and I think the authors should also comment on this potential future criticism. Regarding the M1, I assume that it informs us more about the mother’s mobility also during pregnancy, than the child’s mobility. Again, some additional methodological considerations about the character, potential and limitations of the samples are necessary.

Which tooth was sampled or whether a petrous bone was used is specified for each investigated individual in the supplementary table S1, which shows that most tooth samples were M1 (n=52, which is now also specified in the text), as these were the preferred sample type.

Petrous bone was selected for individuals where the teeth looked altered (e.g. chalky) or were too fragmented. We added this to the text. We also specified that human teeth form in specified time windows in the method section and the problem with comparing different sample types is further explored later on in the ‘Discussion’, particularly under ‘The role of age’.
The densest part of the petrous bone (the otic capsule) has been shown to not only stop remodeling after the age of two, but also to be robust against diagenetic alteration after burial and even against alterations during cremation. That’s why we specified that the densest part of the petrous bone was sampled. We’ve added a statement regarding the suitability of petrous bones under ‘Material’ and cite the following references:

34.	Veselka B, Locher H, de Groot JCMJ, Davies GR, Snoeck C, Kootker LM. Strontium isotope ratios related to childhood mobility: Revisiting sampling strategies of the calcined human pars petrosa ossis temporalis. Rapid Commun Mass Spectrom. 2021;35(7):e9038. doi:10.1002/rcm.9038

35.	Buikstra JE. Standards for data collection from human skeletal remains. Ubelaker DH, editor. Fayeteville, Arkansas: Arkansas Archeological Survey; 1994. 276 p. (Arkansas Archaeological Survey Research Series; no. 44).

This is only true if we are talking about deciduous teeth, which mainly form in utero. However, these were largely excluded from this study and only used for very young sub-adults with no permanent teeth present. Even here, we tried to always sample the deciduous M2, as its formation time post birth is two times longer than pre-birth, thus, the Sr isotope signature of a deciduous M2 should largely reflect the individual’s mobility rather than its mother’s (ignoring the fact, that it is likely that both their travels are likely closely linked until the deciduous M2 completes formation at roughly 10-month-old). While the permanent M1 is the first adult tooth to start forming, it’s formation commences roughly around birth (plus minus some weeks) and does not complete until an individual is 2,5 – 3 years of age. Hence, permanent M1 Sr isotope signatures are representative of an individual’s early childhood diet. We’ve specified this in the ‘Material’ section.

Overall, we have extended our ‘Material’ section to elaborate on the sampling strategy and it read now as follows:

‘A total of 199 inhumated Bronze Age individuals from six archaeological sites in central and southern Greece (Fig 1) were investigated for their childhood origin (The archive IDs for each sample are listed in Table S1). Of these, 55 had been previously analysed for Sr isotopes [2]. The use of this published data is indicated in the sample overview (Table S1). From each individual either a petrous bone (n = 92) or tooth enamel sample (n = 107) was analysed for Sr isotopes, enabling the determination of a potential foreign childhood origin, but not a mobility timeline for the individuals. The human teeth form in a well-established sequence, and their enamel portion does not remodel during an individual’s life or post mortem [8]. Thus, tooth enamel is a reliable archive of an individual’s diet and geographic origin during the crown’s formation making teeth the preferred sample type for this study. Wherever possible, sampling of the first molar was done (n = 52), as its formation commences around an individual’s birth and completes around three years of age, providing a clearly constrained 87Sr/86Sr signature of an individual’s early childhood. While bone samples are generally considered less suitable to determine childhood origin, as they are susceptible to alteration, the petrous bone has been shown to be resistant to remodelling and diagenetic alteration after the age of two [33,34]. Hence, they were considered a suitable Sr isotope archive of an individual’s early childhood, when no suitable teeth were available. Which specific tooth was chosen, or whether a petrous bone was sampled depended ultimately on the preservation of the remains, with altered looking or fragmented samples excluded from this study.

The archaeological material included in this study was provided by the excavation directors of each site. The archive IDs for all samples are listed in Table S1. The permits required for this study were granted by the Greek Ministry of Culture and the study complied with all relevant regulations.’

The overall results are very interesting - a seemingly low percentage of non-local individuals in comparison to other parts of Europe, which might, however, be also due to the overall homogeneity of the bioavailable Sr which presents a strong limitation on the method in present-day Greece. In spite of the limited number of non-local individuals, the authors do their best to contextualise their results in all possible perspectives - age, gender, archaeological context etc. One would have wished that this ambitious study would have had the chance to produce even further historical insights, but in my view it demonstrates how far Sr isotope analysis in Greece can go and what are the inherent potentials and limitations. Therefore, I consider this study very relevant for publication in PlosOne.

Some minor points:
l. 50: which textual evidence informs us about “changes to the local architecture and settlement organization”? I am not aware of any, but if there is one, the authors should provide some further explanation rather than staying so vague.

Thank you for the careful review. We’ve rewritten the introduction and deleted the word ‘textual’ as it was incorrect (see also answer to the first major comment).

l. 55f.: the authors should refer to the evidence from EH II Nea Styra (Skourtanioti et al. 2023), where the genetic evidence identified non-local individuals of Anatolian genetic ancestry, whereas this is not mirrored in the burial goods.

Thank you for the suggestion. We’ve added the study’s finding to our introduction (see also answer to the first major comment).

l. 115: I find scholars’ titles in publications somehow awkward. I suggest deleting the “Prof.”

We deleted the title.

l. 134: same for “Dr.” here

We deleted the title.
