# Supplementary material for: Exploring mobility in Bronze Age Greece: A Sr isotope perspective
Source: PLoS One. 2026 Sep 23;21(9):e0357576. doi: 10.1371/journal.pone.0357576 (PMC13600528; doi:10.1371/journal.pone.0357576)
Supplement: Peer review history

# Peer review: Decision Letter 0

**DOI:** 10.1371/journal.pone.0357576.r001

**Authors:** Stefanos Gimatzidis

9 Jul 2026

Dear Dr. Frank,

While both reviewers are generally positive about your work, Reviewer 2 has raised critical questions regarding your sampling methodology. Accordingly, you are invited to revise your manuscript. Please take both reviewers’ comments into careful consideration and provide a point-by-point response alongside your revised submission.

Please submit your revised manuscript by Aug 07 2026 11:59PM. If you will need more time than this to complete your revisions, please reply to this message or contact the journal office at plosone@plos.org. When you're ready to submit your revision, log on to https://www.editorialmanager.com/pone/ and select the 'Submissions Needing Revision' folder to locate your manuscript file.

- A letter that responds to each point raised by the academic editor and reviewer(s). You should upload this letter as a separate file labeled 'Response to Reviewers'.
- A marked-up copy of your manuscript that highlights changes made to the original version. You should upload this as a separate file labeled 'Revised Manuscript with Track Changes'.
- An unmarked version of your revised paper without tracked changes. You should upload this as a separate file labeled 'Manuscript'.

If you would like to make changes to your financial disclosure, please include your updated statement in your cover letter. Guidelines for resubmitting your figure files are available below the reviewer comments at the end of this letter.

If applicable, we recommend that you deposit your laboratory protocols in protocols.io to enhance the reproducibility of your results. Protocols.io assigns your protocol its own identifier (DOI) so that it can be cited independently in the future. For instructions see: https://journals.plos.org/plosone/s/submission-guidelines#loc-laboratory-protocols. Additionally, PLOS ONE offers an option for publishing peer-reviewed Lab Protocol articles, which describe protocols hosted on protocols.io. Read more information on sharing protocols at https://plos.org/protocols?utm_medium=editorial-email&utm_source=authorletters&utm_campaign=protocols.

As the corresponding author, your ORCID iD is verified in the submission system and will appear in the published article. PLOS supports the use of ORCID, and we encourage all coauthors to register for an ORCID iD and use it as well. Please encourage your coauthors to verify their ORCID iD within the submission system before final acceptance, as unverified ORCID iDs will not appear in the published article. *Only*  the individual author can complete the verification step; PLOS staff *cannot*  verify ORCID iDs on behalf of authors.

We look forward to receiving your revised manuscript.

Kind regards,

Stefanos Gimatzidis, Ph.D.

Academic Editor

PLOS One

Journal Requirements:

When submitting your revision, we need you to address these additional requirements.

1. Please ensure that your manuscript meets PLOS ONE's style requirements, including those for file naming. The PLOS ONE style templates can be found at

https://journals.plos.org/plosone/s/file?id=wjVg/PLOSOne_formatting_sample_main_body.pdf and https://journals.plos.org/plosone/s/file?id=ba62/PLOSOne_formatting_sample_title_authors_affiliations.pdf

2. Please include a complete copy of PLOS’ questionnaire on inclusivity in global research in your revised manuscript. Our policy for research in this area aims to improve transparency in the reporting of research performed outside of researchers’ own country or community. The policy applies to researchers who have travelled to a different country to conduct research, research with Indigenous populations or their lands, and research on cultural artefacts. The questionnaire can also be requested at the journal’s discretion for any other submissions, even if these conditions are not met.  Please find more information on the policy and a link to download a blank copy of the questionnaire here: https://journals.plos.org/plosone/s/best-practices-in-research-reporting. Please upload a completed version of your questionnaire as Supporting Information when you resubmit your manuscript.

3. Please provide a complete Data Availability Statement in the submission form, ensuring you include all necessary access information or a reason for why you are unable to make your data freely accessible. If your research concerns only data provided within your submission, please write "All data are in the manuscript and/or supporting information files" as your Data Availability Statement.

4. We note that the grant information you provided in the ‘Funding Information’ and ‘Financial Disclosure’ sections do not match.

When you resubmit, please ensure that you provide the correct grant numbers for the awards you received for your study in the ‘Funding Information’ section.

5. Thank you for stating the following financial disclosure:

The Sr isotope work of this study was made possible through the funding provided by the “Semper Ardens” research grant CF18-0005 from Carlsbergfondet (KMF; https://www.carlsbergfondet.dk/en) and the Jubileumsfond grant M16-0455 :1 from Sveriges Riksbanken (KK; https://www.rj.se/en/). The bioarchaeological study was supported by the European Union’s Marie Skłodowska-Curie Actions grant No. 747458 (IM; https://marie-sklodowska-curie-actions.ec.europa.eu/), and the Malcolm H. Wiener Laboratory for Archaeological Science Post-doctoral Research Fellowship (IM; https://www.ascsa.edu.gr/research/wiener-laboratory).

Please state what role the funders took in the study. If the funders had no role, please state: "The funders had no role in study design, data collection and analysis, decision to publish, or preparation of the manuscript."

If this statement is not correct you must amend it as needed.

Please include this amended Role of Funder statement in your cover letter; we will change the online submission form on your behalf.

6. We note that Figure 1 in your submission contain [map/satellite] images which may be copyrighted. All PLOS content is published under the Creative Commons Attribution License (CC BY 4.0), which means that the manuscript, images, and Supporting Information files will be freely available online, and any third party is permitted to access, download, copy, distribute, and use these materials in any way, even commercially, with proper attribution. For these reasons, we cannot publish previously copyrighted maps or satellite images created using proprietary data, such as Google software (Google Maps, Street View, and Earth). For more information, see our copyright guidelines: http://journals.plos.org/plosone/s/licenses-and-copyright.

We require you to either (1) present written permission from the copyright holder to publish these figures specifically under the CC BY 4.0 license, or (2) remove the figures from your submission:

a. You may seek permission from the original copyright holder of Figure 1 to publish the content specifically under the CC BY 4.0 license.

We recommend that you contact the original copyright holder with the Content Permission Form (http://journals.plos.org/plosone/s/file?id=7c09/content-permission-form.pdf) and the following text:

“I request permission for the open-access journal PLOS ONE to publish XXX under the Creative Commons Attribution License (CCAL) CC BY 4.0 (http://creativecommons.org/licenses/by/4.0/). Please be aware that this license allows unrestricted use and distribution, even commercially, by third parties. Please reply and provide explicit written permission to publish XXX under a CC BY license and complete the attached form.”

Please upload the completed Content Permission Form or other proof of granted permissions as an "Other" file with your submission.

In the figure caption of the copyrighted figure, please include the following text: “Reprinted from [ref] under a CC BY license, with permission from [name of publisher], original copyright [original copyright year].”

b. If you are unable to obtain permission from the original copyright holder to publish these figures under the CC BY 4.0 license or if the copyright holder’s requirements are incompatible with the CC BY 4.0 license, please either i) remove the figure or ii) supply a replacement figure that complies with the CC BY 4.0 license. Please check copyright information on all replacement figures and update the figure caption with source information. If applicable, please specify in the figure caption text when a figure is similar but not identical to the original image and is therefore for illustrative purposes only.

The following resources for replacing copyrighted map figures may be helpful:

USGS National Map Viewer (public domain): http://viewer.nationalmap.gov/viewer/

The Gateway to Astronaut Photography of Earth (public domain): http://eol.jsc.nasa.gov/sseop/clickmap/

Maps at the CIA (public domain): https://www.cia.gov/library/publications/the-world-factbook/index.html and https://www.cia.gov/library/publications/cia-maps-publications/index.html

NASA Earth Observatory (public domain): http://earthobservatory.nasa.gov/

Landsat: http://landsat.visibleearth.nasa.gov/

USGS EROS (Earth Resources Observatory and Science (EROS) Center) (public domain): http://eros.usgs.gov/#

Natural Earth (public domain): http://www.naturalearthdata.com/

7. Please include a copy of Table 3, which you refer to in your text on page 8, 9.

8. We note that there is identifying data in the Supporting Information file <PlosONE_Greece_tablesS1_1.xlsx>. Due to the inclusion of these potentially identifying data, we have removed this file from your file inventory. Prior to sharing human research participant data, authors should consult with an ethics committee to ensure data are shared in accordance with participant consent and all applicable local laws.

Data sharing should never compromise participant privacy. It is therefore not appropriate to publicly share personally identifiable data on human research participants. The following are examples of data that should not be shared:

-Name, initials, physical address

-Ages more specific than whole numbers

-Internet protocol (IP) address

-Specific dates (birth dates, death dates, examination dates, etc.)

-Contact information such as phone number or email address

-Location data

-ID numbers that seem specific (long numbers, include initials, titled “Hospital ID”) rather than random (small numbers in numerical order)

Data that are not directly identifying may also be inappropriate to share, as in combination they can become identifying. For example, data collected from a small group of participants, vulnerable populations, or private groups should not be shared if they involve indirect identifiers (such as sex, ethnicity, location, etc.) that may risk the identification of study participants.

Additional guidance on preparing raw data for publication can be found in our Data Policy (https://journals.plos.org/plosone/s/data-availability#loc-human-research-participant-data-and-other-sensitive-data) and in the following article: http://www.bmj.com/content/340/bmj.c181.long.

Please remove or anonymize all personal information (<specific identifying information in file to be removed>), ensure that the data shared are in accordance with participant consent, and re-upload a fully anonymized data set. Please note that spreadsheet columns with personal information must be removed and not hidden as all hidden columns will appear in the published file.

9. If the reviewer comments include a recommendation to cite specific previously published works, please review and evaluate these publications to determine whether they are relevant and should be cited. There is no requirement to cite these works unless the editor has indicated otherwise.

10. Please review your reference list to ensure that it is complete and correct. If you have cited papers that have been retracted, please include the rationale for doing so in the manuscript text, or remove these references and replace them with relevant current references. Any changes to the reference list should be mentioned in the rebuttal letter that accompanies your revised manuscript. If you need to cite a retracted article, indicate the article’s retracted status in the References list and also include a citation and full reference for the retraction notice.

[Note: HTML markup is below. Please do not edit.]

Reviewers' comments:

Reviewer's Responses to Questions

**Comments to the Author**

1. Is the manuscript technically sound, and do the data support the conclusions?

Reviewer #1: Yes

Reviewer #2: Yes

**********

2. Has the statistical analysis been performed appropriately and rigorously?

Reviewer #1: Yes

Reviewer #2: Yes

**********

3. Have the authors made all data underlying the findings in their manuscript fully available?

The PLOS Data policy

Reviewer #1: Yes

Reviewer #2: Yes

**********

4. Is the manuscript presented in an intelligible fashion and written in standard English?

Reviewer #1: Yes

Reviewer #2: Yes

**********

Reviewer #1: Based on a data base of samples of the skeletal remains of 199 individuals from six archaeological sites and due to a carefully designed mathodological approach, the article constitutes an important addition to our knowledge about applying the method of Sr-isotopy to gain insights into issues of mobility in Bronze Age Greece. The authors demonstrate that, if applied to human remains in Greece, the results of this method are far less equivocal than in other parts of the world, because, due to similar geological circumstances, the Sr-baselines of many Greek regions overlap to a certain degree. To counter these problems, the authors have refined their approach by combining a Sr-baseline based on the geological data available for the surroundings of a site with a site-specific human baseline. In this way, they are able to mitigate the problem of overlapping baselines which allows them to define a certain number of non-local people in the respective data sets of the investigated sites. Their calculation of percentages between 5 and 10% of non-locals throughout the Bronze Age should be regarded as a minimum, since, as the authors stress, there must be many non-locals that remain undetected due to the still existing significant overlap of Sr-baseline between various regions of Greece and even other parts of the Mediterranean. Important are also the conclusions that the identified non-locals were predominantly young adults and that these patterns of mobility do not seem to have been gender-specific.

In the general, the manuscript is carefully written, but I found a small number of spelling mistakes:

- p. 25 "Southern Germany" (not "Southern German");

- p. 25: "as well as a in a LBA community" (not: "as well as a in LBA community");

- p. 30: "only 17 have....signatures") (not: "only 17 have.....signature").

Reviewer #2: With their article, Frank et al. fill a most important gap of bioarchaeological research in Greece, namely Strontium isotope data. The last years have seen an increasing number of other bioarchaeological datasets, especially in the field of archaeogenetics, but Strontium data have been scarce. The publication of 199 skeletal samples, therefore, presents an enormous step forward.

I think that the article could profit from some more precision through. This is particularly when the authors speak about the “Bronze Age”. When they describe the Bronze Age, e.g. “saw rapid economic and cultural changes” (l. 28) and that “Greece was a centre for economic and cultural innovation” (l. 48-49) (whereby I was asking myself, which cultural innovation the authors refer to…). Whereas this is true for some parts of the Bronze Age, this can definitely not be said for the whole period from 3000 to 1050 BC with all its non-linear developments, social transformations and regional specificities. During the Middle Bronze Age, Crete was indeed an economic and cultural center, whereas the Mainland was far from that etc. Here, I think the authors should avoid such overarching generalisations about THE “Greek Bronze Age” and be more specific in a spatio-temporal perspective when they refer to cultural topics.

As it is stated in the materials chapter, the study included teeth and petrous bones for Sr analysis. Here, I think it would have been essential to further elaborate on the sampling strategy. What is the share of the different molars among the samples (M1, M2, M3)? The authors just state that M1 and M2 were preferred. Why did they select petrous bones instead of teeth in some cases and what was the sampling strategy behind? The reviewer is aware of the fragmentary preservation of skeletal material, especially in the Bronze Age collective burials of the Aegean. However, I think it is necessary to elaborate on the inherent challenges of sampling and comparing different types of samples as well as different molars and how this influences the results. Moreover, some scholars have argued that the petrous portion might take up Sr isotopes from the soil post-mortem and I think the authors should also comment on this potential future criticism. Regarding the M1, I assume that it informs us more about the mother’s mobility also during pregnancy, than the child’s mobility. Again, some additional methodological considerations about the character, potential and limitations of the samples are necessary.

The overall results are very interesting - a seemingly low percentage of non-local individuals in comparison to other parts of Europe, which might, however, be also due to the overall homogeneity of the bioavailable Sr which presents a strong limitation on the method in present-day Greece. In spite of the limited number of non-local individuals, the authors do their best to contextualise their results in all possible perspectives - age, gender, archaeological context etc. One would have wished that this ambitious study would have had the chance to produce even further historical insights, but in my view it demonstrates how far Sr isotope analysis in Greece can go and what are the inherent potentials and limitations. Therefore, I consider this study very relevant for publication in PlosOne.

Some minor points:

l. 50: which textual evidence informs us about “changes to the local architecture and settlement organization”? I am not aware of any, but if there is one, the authors should provide some further explanation rather than staying so vague.

l. 55f.: the authors should refer to the evidence from EH II Nea Styra (Skourtanioti et al. 2023), where the genetic evidence identified non-local individuals of Anatolian genetic ancestry, whereas this is not mirrored in the burial goods.

l. 115: I find scholars’ titles in publications somehow awkward. I suggest deleting the “Prof.”

l. 134: same for “Dr.” here

**********

what does this mean?). If published, this will include your full peer review and any attached files.

If you choose “no”, your identity will remain anonymous but your review may still be made public.

**Do you want your identity to be public for this peer review?**  For information about this choice, including consent withdrawal, please see our Privacy Policy

Reviewer #1: No

Reviewer #2: No

**********

[NOTE: If reviewer comments were submitted as an attachment file, they will be attached to this email and accessible via the submission site. Please log into your account, locate the manuscript record, and check for the action link "View Attachments". If this link does not appear, there are no attachment files.]

To ensure your figures meet our technical requirements, please review our figure guidelines: https://journals.plos.org/plosone/s/figures

You may also use PLOS’s free figure tool, NAAS, to help you prepare publication quality figures: https://journals.plos.org/plosone/s/figures#loc-tools-for-figure-preparation.

NAAS will assess whether your figures meet our technical requirements by comparing each figure against our figure specifications.

# Author comment: Author response to Decision Letter 1

**DOI:** 10.1371/journal.pone.0357576.r002

6 Aug 2026

Dear Editor,

Thank you for considering our manuscript titled ‘Exploring mobility in Bronze Age Greece: A Sr isotope perspective’ for publication in PlosONE. We’ve copied the outlined journal requirements and reviewer comments in black below and added how we addressed these in blue.

Journal Requirements:

When submitting your revision, we need you to address these additional requirements.

1. Please ensure that your manuscript meets PLOS ONE's style requirements, including those for file naming. The PLOS ONE style templates can be found at

https://journals.plos.org/plosone/s/file?id=wjVg/PLOSOne_formatting_sample_main_body.pdf and https://journals.plos.org/plosone/s/file?id=ba62/PLOSOne_formatting_sample_title_authors_affiliations.pdf

We’ve ensured that our article meets the journal requirements.

2. Please include a complete copy of PLOS’ questionnaire on inclusivity in global research in your revised manuscript. Our policy for research in this area aims to improve transparency in the reporting of research performed outside of researchers’ own country or community. The policy applies to researchers who have travelled to a different country to conduct research, research with Indigenous populations or their lands, and research on cultural artefacts. The questionnaire can also be requested at the journal’s discretion for any other submissions, even if these conditions are not met. Please find more information on the policy and a link to download a blank copy of the questionnaire here: https://journals.plos.org/plosone/s/best-practices-in-research-reporting. Please upload a completed version of your questionnaire as Supporting Information when you resubmit your manuscript.

We’ve filled out and included the questionnaire for our resubmission.

3. Please provide a complete Data Availability Statement in the submission form, ensuring you include all necessary access information or a reason for why you are unable to make your data freely accessible. If your research concerns only data provided within your submission, please write "All data are in the manuscript and/or supporting information files" as your Data Availability Statement.

All data are in the manuscript and/or supporting information files.

4. We note that the grant information you provided in the ‘Funding Information’ and ‘Financial Disclosure’ sections do not match.

When you resubmit, please ensure that you provide the correct grant numbers for the awards you received for your study in the ‘Funding Information’ section.

We’ve amended our financial disclosure to align it with the funding information:

The Sr isotope work of this study was made possible through the funding provided by the “Semper Ardens” research grant CF18-0005 from Carlsbergfondet (KMF; https://www.carlsbergfondet.dk/en) and the Jubileumsfond grant M16-0455 :1 from Sveriges Riksbanken (KK; https://www.rj.se/en/). The bioarchaeological study was supported by the European Union’s Marie Skłodowska-Curie Actions grant No. 747458 (IM; https://marie-sklodowska-curie-actions.ec.europa.eu/). The funders had no role in study design, data collection and analysis, decision to publish, or preparation of the manuscript.

5. Thank you for stating the following financial disclosure:

The Sr isotope work of this study was made possible through the funding provided by the “Semper Ardens” research grant CF18-0005 from Carlsbergfondet (KMF; https://www.carlsbergfondet.dk/en) and the Jubileumsfond grant M16-0455 :1 from Sveriges Riksbanken (KK; https://www.rj.se/en/). The bioarchaeological study was supported by the European Union’s Marie Skłodowska-Curie Actions grant No. 747458 (IM; https://marie-sklodowska-curie-actions.ec.europa.eu/), and the Malcolm H. Wiener Laboratory for Archaeological Science Post-doctoral Research Fellowship (IM; https://www.ascsa.edu.gr/research/wiener-laboratory).

Please state what role the funders took in the study. If the funders had no role, please state: "The funders had no role in study design, data collection and analysis, decision to publish, or preparation of the manuscript."

If this statement is not correct you must amend it as needed.

Please include this amended Role of Funder statement in your cover letter; we will change the online submission form on your behalf.

We’ve amended our statement to include that the funders had no role in study design, data collection and analysis, decision to publish, or preparation of the manuscript (also see comment above).

6. We note that Figure 1 in your submission contain [map/satellite] images which may be copyrighted. All PLOS content is published under the Creative Commons Attribution License (CC BY 4.0), which means that the manuscript, images, and Supporting Information files will be freely available online, and any third party is permitted to access, download, copy, distribute, and use these materials in any way, even commercially, with proper attribution. For these reasons, we cannot publish previously copyrighted maps or satellite images created using proprietary data, such as Google software (Google Maps, Street View, and Earth). For more information, see our copyright guidelines: http://journals.plos.org/plosone/s/licenses-and-copyright.

We require you to either (1) present written permission from the copyright holder to publish these figures specifically under the CC BY 4.0 license, or (2) remove the figures from your submission:

a. You may seek permission from the original copyright holder of Figure 1 to publish the content specifically under the CC BY 4.0 license.

We recommend that you contact the original copyright holder with the Content Permission Form (http://journals.plos.org/plosone/s/file?id=7c09/content-permission-form.pdf) and the following text:

“I request permission for the open-access journal PLOS ONE to publish XXX under the Creative Commons Attribution License (CCAL) CC BY 4.0 (http://creativecommons.org/licenses/by/4.0/). Please be aware that this license allows unrestricted use and distribution, even commercially, by third parties. Please reply and provide explicit written permission to publish XXX under a CC BY license and complete the attached form.”

Please upload the completed Content Permission Form or other proof of granted permissions as an "Other" file with your submission.

In the figure caption of the copyrighted figure, please include the following text: “Reprinted from [ref] under a CC BY license, with permission from [name of publisher], original copyright [original copyright year].”

b. If you are unable to obtain permission from the original copyright holder to publish these figures under the CC BY 4.0 license or if the copyright holder’s requirements are incompatible with the CC BY 4.0 license, please either i) remove the figure or ii) supply a replacement figure that complies with the CC BY 4.0 license. Please check copyright information on all replacement figures and update the figure caption with source information. If applicable, please specify in the figure caption text when a figure is similar but not identical to the original image and is therefore for illustrative purposes only.

The following resources for replacing copyrighted map figures may be helpful:

USGS National Map Viewer (public domain): http://viewer.nationalmap.gov/viewer/

The Gateway to Astronaut Photography of Earth (public domain): http://eol.jsc.nasa.gov/sseop/clickmap/

Maps at the CIA (public domain): https://www.cia.gov/library/publications/the-world-factbook/index.html and https://www.cia.gov/library/publications/cia-maps-publications/index.html

NASA Earth Observatory (public domain): http://earthobservatory.nasa.gov/

Landsat: http://landsat.visibleearth.nasa.gov/

USGS EROS (Earth Resources Observatory and Science (EROS) Center) (public domain): http://eros.usgs.gov/#

Natural Earth (public domain): http://www.naturalearthdata.com/

The map was created from scratch with no copyrighted imagery and can thus be published under CC BY 4.0 without any citations.

7. Please include a copy of Table 3, which you refer to in your text on page 8, 9.

Sorry for this oversight, these citations were from an earlier draft and were, therefore, deleted.

8. We note that there is identifying data in the Supporting Information file <PlosONE_Greece_tablesS1_1.xlsx>. Due to the inclusion of these potentially identifying data, we have removed this file from your file inventory. Prior to sharing human research participant data, authors should consult with an ethics committee to ensure data are shared in accordance with participant consent and all applicable local laws.

Data sharing should never compromise participant privacy. It is therefore not appropriate to publicly share personally identifiable data on human research participants. The following are examples of data that should not be shared:

-Name, initials, physical address

-Ages more specific than whole numbers

-Internet protocol (IP) address

-Specific dates (birth dates, death dates, examination dates, etc.)

-Contact information such as phone number or email address

-Location data

-ID numbers that seem specific (long numbers, include initials, titled “Hospital ID”) rather than random (small numbers in numerical order)

Data that are not directly identifying may also be inappropriate to share, as in combination they can become identifying. For example, data collected from a small group of participants, vulnerable populations, or private groups should not be shared if they involve indirect identifiers (such as sex, ethnicity, location, etc.) that may risk the identification of study participants.

Additional guidance on preparing raw data for publication can be found in our Data Policy (https://journals.plos.org/plosone/s/data-availability#loc-human-research-participant-data-and-other-sensitive-data) and in the following article: http://www.bmj.com/content/340/bmj.c181.long.

Please remove or anonymize all personal information (<specific identifying information in file to be removed>), ensure that the data shared are in accordance with participant consent, and re-upload a fully anonymized data set. Please note that spreadsheet columns with personal information must be removed and not hidden as all hidden columns will appear in the published file.

There is no identifying data included in Table S1. The investigated individuals lived and died thousands of years ago and their identities have been lost to history. The included Archive IDs are the assigned IDs to the sampled teeth and bones (not full individuals) and are included to help future scientists interested in conducting further research to identify and find the samples in question. This was done to adhere to the FAIR principles.

9. If the reviewer comments include a recommendation to cite specific previously published works, please review and evaluate these publications to determine whether they are relevant and should be cited. There is no requirement to cite these works unless the editor has indicated otherwise.

The only suggested reference was a prehistoric Aegean DNA study, that was already included in the study. Thus, it is appropriate for the study and we added a sentence/citation expanding on the study’s findings.

10. Please review your reference list to ensure that it is complete and correct. If you have cited papers that have been retracted, please include the rationale for doing so in the manuscript text, or remove these references and replace them with relevant current references. Any changes to the reference list should be mentioned in the rebuttal letter that accompanies your revised manuscript. If you need to cite a retracted article, indicate the article’s retracted status in the References list and also include a citation and full reference for the retraction notice.

We could not find any issues with our reference list. We’ve added only two references to address the concerns of Reviewer 2 regarding the suitability of petrous bones (see comments below).

Comments to the Author

Reviewer #1: Based on a data base of samples of the skeletal remains of 199 individuals from six archaeological sites and due to a carefully designed mathodological approach, the article constitutes an important addition to our knowledge about applying the method of Sr-isotopy to gain insights into issues of mobility in Bronze Age Greece. The authors demonstrate that, if applied to human remains in Greece, the results of this method are far less equivocal than in other parts of the world, because, due to similar geological circumstances, the Sr-baselines of many Greek regions overlap to a certain degree. To counter these problems, the authors have refined their approach by combining a Sr-baseline based on the geological data available for the surroundings of a site with a site-specific human baseline. In this way, they are able to mitigate the problem of overlapping baselines which allows them to define a certain number of non-local people in the respective data sets of the investigated sites. Their calculation of percentages between 5 and 10% of non-locals throughout the Bronze Age should be regarded as a minimum, since, as the authors stress, there must be many non-locals that remain undetected due to the still existing significant overlap of Sr-baseline between various regions of Greece and even other parts of the Mediterranean. Important are also the conclusions that the identified non-locals were predominantly young adults and that these patterns of mobility do not seem to have been gender-specific.

In the general, the manuscript is carefully written, but I found a small number of spelling mistakes:

- p. 25 "Southern Germany" (not "Southern German");

- p. 25: "as well as a in a LBA community" (not: "as well as a in LBA community");

- p. 30: "only 17 have....signatures") (not: "only 17 have.....signature").

Thank you for the careful review. We’ve made the necessary adjustments.

Reviewer #2: With their article, Frank et al. fill a most important gap of bioarchaeological research in Greece, namely Strontium isotope data. The last years have seen an increasing number of other bioarchaeological datasets, especially in the field of archaeogenetics, but Strontium data have been scarce. The publication of 199 skeletal samples, therefore, presents an enormous step forward.

I think that the article could profit from some more precision through. This is particularly when the authors speak about the “Bronze Age”. When they describe the Bronze Age, e.g. “saw rapid economic and cultural changes” (l. 28) and that “Greece was a centre for economic and cultural innovation” (l. 48-49) (whereby I was asking myself, which cultural innovation the authors refer to…). Whereas this is true for some parts of the Bronze Age, this can definitely not be said for the whole period from 3000 to 1050 BC with all its non-linear developments, social transformations and regional specificities. During the Middle Bronze Age, Crete was indeed an economic and cultural center, whereas the Mainland was far from that etc. Here, I think the authors should avoid such overarching generalisations about THE “Greek Bronze Age” and be more specific in a spatio-temporal perspective when they refer to cultural topics.

We’ve amended the starting sentence to the Abstract (l.28) to: ‘The Mediterranean region saw significant socio-economic and cultural transformations during the Bronze Age, varying across regions and through time. Expanding exchange networks played an important role in these developments by connecting distant communities.’, to introduce the variability in the mentioned economic and cultural developments.

Further we extended our introduction (originally l. 48-59) to elaborate further. It now reads as follows:

‘During the Bronze Age, the region of modern-day Greece underwent substantial social, economic, and cultural transformations, differently expressed across regions and through time. In particular, the transition from the Middle to Late Bronze Age witnessed the formation of Mycenaean societies on the mainland, accompanied by increasing socio-political complexity, expanding exchange networks, and significant changes in settlement organisation and material culture. Following diverse local trajectories, these developments were crucial for the e

Attachment Submitted filename: PlosONE_Greece_response.docx

# Editor report: Decision Letter 1

**DOI:** 10.1371/journal.pone.0357576.r003

**Authors:** Stefanos Gimatzidis

20 Aug 2026

Exploring mobility in Bronze Age Greece: A Sr isotope perspective

PONE-D-26-23037R1

Dear Dr. Frank,

We’re pleased to inform you that your manuscript has been judged scientifically suitable for publication and will be formally accepted for publication once it meets all outstanding technical requirements.

Within one week, you’ll receive an e-mail detailing the required amendments. When these have been addressed, you’ll receive a formal acceptance letter and your manuscript will be scheduled for publication.

An invoice will be generated when your article is formally accepted. Please note, if your institution has a publishing partnership with PLOS and your article meets the relevant criteria, all or part of your publication costs will be covered. Please make sure your user information is up-to-date by logging into Editorial Manager at Editorial Manager® and clicking the ‘Update My Information' link at the top of the page. For questions related to billing, please contact billing support.

If your institution or institutions have a press office, please notify them about your upcoming paper to help maximize its impact. If they’ll be preparing press materials, please inform our press team as soon as possible -- no later than 48 hours after receiving the formal acceptance. Your manuscript will remain under strict press embargo until 2 pm Eastern Time on the date of publication. For more information, please contact onepress@plos.org.

Kind regards,

Stefanos Gimatzidis, Ph.D.

Academic Editor

PLOS One

Additional Editor Comments (optional):

Reviewers' comments:

# Editor report: Acceptance letter

**DOI:** 10.1371/journal.pone.0357576.r004

**Authors:** Stefanos Gimatzidis

PONE-D-26-23037R1

PLOS One

Dear Dr. Frank,

I'm pleased to inform you that your manuscript has been deemed suitable for publication in PLOS One. Congratulations! Your manuscript is now being handed over to our production team.

At this stage, our production department will prepare your paper for publication. This includes ensuring the following:

* All references, tables, and figures are properly cited

* All relevant supporting information is included in the manuscript submission,

* There are no issues that prevent the paper from being properly typeset

You will receive further instructions from the production team, including instructions on how to review your proof when it is ready. Please keep in mind that we are working through a large volume of accepted articles, so please give us a few days to review your paper and let you know the next and final steps.

Lastly, if your institution or institutions have a press office, please let them know about your upcoming paper now to help maximize its impact. If they'll be preparing press materials, please inform our press team within the next 48 hours. Your manuscript will remain under strict press embargo until 2 pm Eastern Time on the date of publication. For more information, please contact onepress@plos.org.

You will receive an invoice from PLOS for your publication fee after your manuscript has reached the completed accept phase. If you receive an email requesting payment before acceptance or for any other service, this may be a phishing scheme. Learn how to identify phishing emails and protect your accounts at https://explore.plos.org/phishing.

If we can help with anything else, please email us at customercare@plos.org.

Thank you for submitting your work to PLOS One and supporting open access.

Kind regards,

PLOS One Editorial Office Staff

on behalf of

Dr. Stefanos Gimatzidis

Academic Editor

PLOS One
